# Electronic cooling via interlayer Coulomb coupling in multilayer epitaxial graphene

**DOI:** 10.1038/ncomms9105

**Published:** 2015-09-24

**Authors:** Momchil T. Mihnev, John R. Tolsma, Charles J. Divin, Dong Sun, Reza Asgari, Marco Polini, Claire Berger, Walt A. de Heer, Allan H. MacDonald, Theodore B. Norris

**Affiliations:** 1 Department of Electrical Engineering and Computer Science, University of Michigan, Ann Arbor, Michigan 48109, USA; 2 Center for Ultrafast Optical Science, University of Michigan, Ann Arbor, Michigan 48109, USA; 3 Department of Physics, The University of Texas at Austin, Austin, Texas 78712, USA; 4 International Center for Quantum Materials, School of Physics, Peking University, Beijing 100871, China; 5 School of Physics, Institute for Research in Fundamental Sciences (IPM), Tehran 19395-5531, Iran; 6 NEST, Istituto Nanoscienze-CNR and Scuola Normale Superiore, Pisa I-56126, Italy; 7 Istituto Italiano di Tecnologia, Graphene Labs, Via Morego 30, Genova I-16163, Italy; 8 School of Physics, Georgia Institute of Technology, Atlanta, Georgia 30332, USA; 9 Institut Neel, CNRS UJF-INP, Grenoble 38042, France; 10 King Abdulaziz University, Jeddah 22254, Saudi Arabia

## Abstract

In van der Waals bonded or rotationally disordered multilayer stacks of two-dimensional (2D) materials, the electronic states remain tightly confined within individual 2D layers. As a result, electron–phonon interactions occur primarily within layers and interlayer electrical conductivities are low. In addition, strong covalent in-plane intralayer bonding combined with weak van der Waals interlayer bonding results in weak phonon-mediated thermal coupling between the layers. We demonstrate here, however, that Coulomb interactions between electrons in different layers of multilayer epitaxial graphene provide an important mechanism for interlayer thermal transport, even though all electronic states are strongly confined within individual 2D layers. This effect is manifested in the relaxation dynamics of hot carriers in ultrafast time-resolved terahertz spectroscopy. We develop a theory of interlayer Coulomb coupling containing no free parameters that accounts for the experimentally observed trends in hot-carrier dynamics as temperature and the number of layers is varied.

## Abstract (web summary)

The coupling between layers plays an important role in the properties of stacked two-dimensional materials. Here, the authors show that Coulomb interactions between electrons in different layers of graphene induce thermal transport even though all electronic states are confined to individual layers.

## Graphical abstract

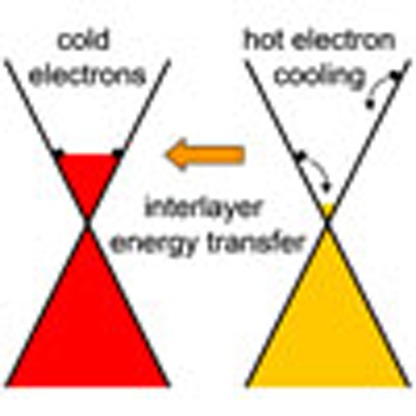

The dynamics of electrons in atomic-layer two-dimensional (2D) electron systems such as graphene is a subject of considerable current interest, partly because of its relevance to a wide variety of potential electronic and optoelectronic device applications. Many proposed and prototype devices employ stacks composed of many 2D electronic material layers. Examples of multilayer systems include multilayer epitaxial graphene (MEG)^1^,^2^, van der Waals bonded layered sheets^3^,^4^, transition metal dichalcogenides^5^ and others^6^. In these structures, the interactions between electrons in different layers in the stack becomes a subject of key importance. One important property is that phonon-mediated interlayer thermal coupling is weak relative to that in bulk three-dimensional (3D) materials. In the example of MEG, rotational stacking arrangements decouple electronic states localized in different 2D layers^7^,^8^. As a result, phonon-mediated interlayer thermal coupling in MEG is strongly reduced relative to typical bulk behaviour in 3D materials.

The question thus arises as to what other mechanisms can contribute to thermal equilibration between different layers. We consider this question here in the context of hot-carrier dynamics. If electrons are heated in one layer (for example, by optical excitation or electrical injection), they will normally cool to the lattice temperature by optical phonon emission at high carrier energies, and by acoustic phonon emission at low carrier energies. For graphene, it is well established that electronic cooling by acoustic phonons is very efficient in highly doped layers^9^,^10^,^11^. The situation is quite different, however, for lightly doped or nearly neutral graphene in which a small joint-density-of-states for electronic transitions combines with a small acoustic phonon energy at typical scattering wavevectors to diminish the acoustic phonon cooling power^10^,^11^. (The cooling power in this limit can, however, be substantially enhanced by disorder, because it relaxes momentum conservation limits^12^,^13^,^14^ on allowed processes).

An interesting case thus arises when a multilayer stack contains both highly doped (high-density (HD)) and nearly neutral lightly doped (low-density (LD)) graphene layers. This is exactly the situation that occurs in MEG grown on the C-face of SiC^1^,^2^, and is likely to be relevant to gated multilayer 2D systems due to interlayer screening^15^,^16^. If electrons are heated in the multilayer structure, then acoustic-phonon-mediated cooling would result in the rapid buildup of a thermal gradient between the HD and LD layers; the HD layers would quickly approach the lattice temperature, while carriers in the LD layers would remain hot. Eventually, of course, thermal equilibrium would be restored, as thermal energy flows from the LD to the HD layers. The HD layers can be a heat sink for the LD layers if there is an effective interlayer energy transfer mechanism.

In the following, we show that Coulomb scattering between electrons in LD and HD layers of MEG can provide an efficient means for interlayer thermal coupling, and provide an alternate mechanism for cooling of hot electrons in the LD layers that acts in parallel with acoustic-phonon-mediated intralayer cooling. This process is illustrated schematically in Fig. 1a. We note that interlayer thermal coupling via Coulomb scattering has been considered recently in the context of 2D electron gases in transport devices^17^,^18^. We begin by outlining a heuristic analytical model calculation for a pair of graphene layers to establish the magnitude of the effect relative to acoustic-phonon-mediated intralayer cooling. This simple calculation establishes the significance of the effect; we then discuss how hot-carrier cooling in multilayer systems is accessible experimentally by ultrafast time-resolved terahertz (THz) spectroscopy and ultrafast infrared (IR) pump–probe spectroscopy measurements. Following a discussion of the features of the data that point to interlayer energy transfer, we present the details of a theory of interlayer energy transfer via screened Coulomb interactions. The calculated cooling powers imply asymptotic cooling times on the sub-nanosecond scale. We show that the calculated dynamics and trends with lattice temperature and number of epitaxial graphene layers are fully consistent with the experimental results, without the need for any fitting parameters.

## Results

### Interlayer Coulombic energy transfer heuristics

**Figure 1:**
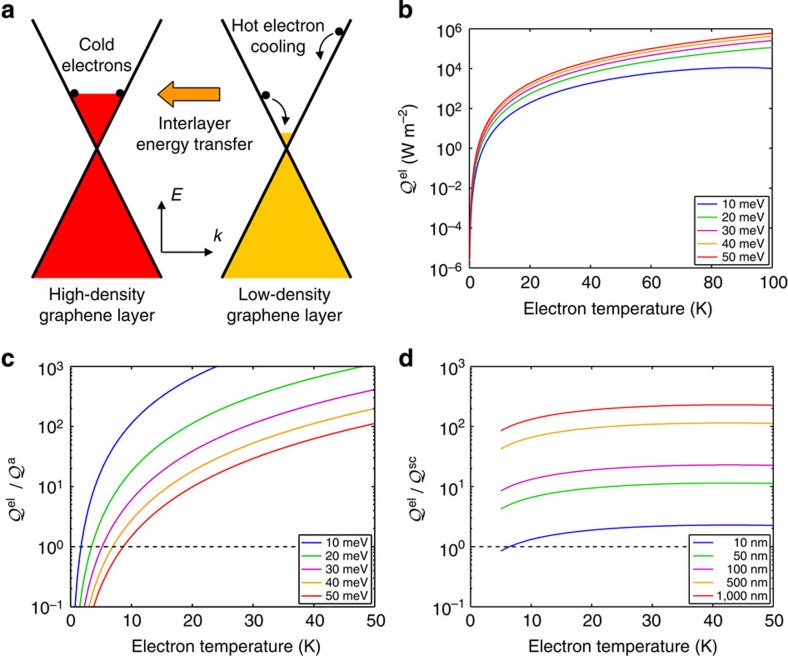
Interlayer Coulombic energy transfer between two graphene layers. (**a**) Schematic diagram of interlayer Coulombic energy transfer from a hot LD to a cold HD graphene layer. (**b**) Cooling power of interlayer Coulombic energy transfer 
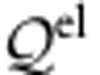
 as a function of electron temperature for Fermi level *E*_F,HD_=300 meV in the HD graphene layer and various Fermi levels *E*_F,LD_ in the LD graphene layer. (**c**) Ratio of the cooling power of interlayer Coulombic energy transfer 
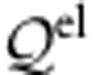
 to the cooling power of intralayer acoustic phonon cooling 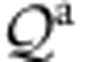 as a function of electron temperature for *E*_F,HD_=300 meV, and various values of *E*_F,LD_ showing that interlayer Coulombic energy transfer can dominate intralayer acoustic phonon cooling in the LD graphene layer. (**d**) Ratio of the cooling power of interlayer Coulombic energy transfer 
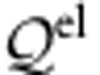
 to the cooling power of intralayer disorder-assisted electron–phonon (supercollision) cooling 
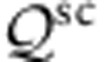
 as a function of electron temperature for *E*_F,HD_=300 meV, *E*_F,LD_=10 meV (typical for C-face MEG on SiC) and various values of the disorder mean free path in the LD graphene layer showing that interlayer Coulombic energy transfer can dominate intralayer disorder-assisted electron–phonon (supercollision) cooling in the LD graphene layer.

We first ask whether or not interlayer Coulomb coupling can potentially dominate over acoustic phonon cooling^10^ and disorder-assisted electron–phonon (supercollision) cooling^14^ in multilayer graphene samples. A simple comparison of the cooling powers of the different mechanisms suggests that the answer is yes. The low-temperature cooling power 
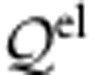
 of interlayer Coulombic energy transfer between a hot LD and a cold HD graphene layer is as follows (see Supplementary Notes 2 and 3):


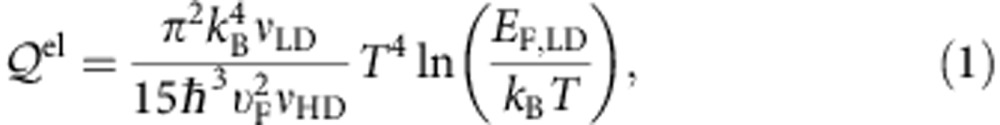


where 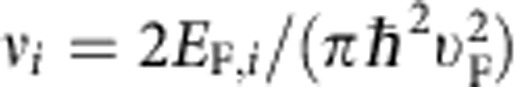 is the density of states at the Fermi level. Equation (1) is plotted in Fig. 1b as a function of electron temperature for the Fermi level *E*_F,HD_=300 meV in the HD layer and various Fermi levels *E*_F,LD_ in the LD layer; even at very low carrier density in the LD layer, the cooling power is quite substantial. We can compare equation (1) with the cooling powers of both acoustic phonon cooling 
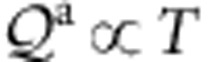
 and disorder-assisted electron–phonon (supercollision) cooling 
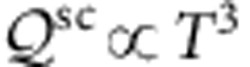
 of an isolated LD layer. The ratio of cooling powers 
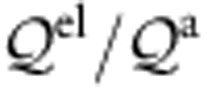
 is plotted in Fig. 1c as a function of electron temperature for *E*_F,HD_=300 meV and various values of *E*_F,LD_. As it can be expected, acoustic phonon cooling is very inefficient in graphene with very low carrier density. The ratio of cooling powers 
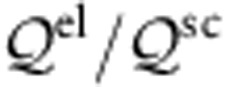
 is also plotted in Fig. 1d as a function of electron temperature (above the Bloch–Grüneisen temperature *T*_BG_≈5 K) for *E*_F,HD_=300 meV, *E*_F,LD_=10 meV (typical for C-face MEG on SiC) and various values of the LD-layer disorder mean free path. It is apparent that for high-quality graphene such as C-face MEG on SiC^19^, interlayer Coulombic energy transfer can dominate for a wide range of electron temperatures and sample characteristics.

### Multilayer epitaxial graphene

**Figure 2:**
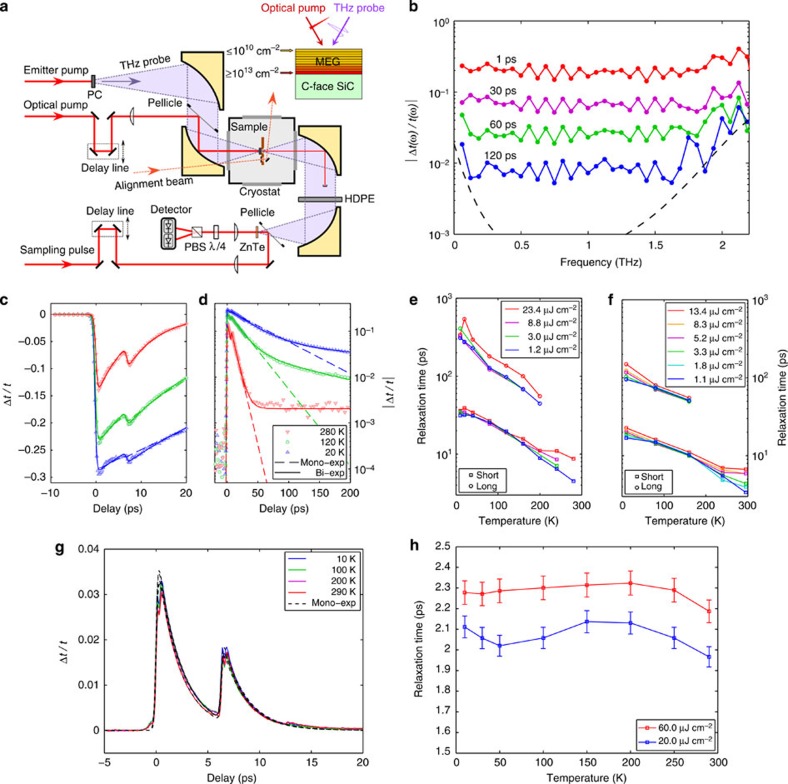
Ultrafast time-resolved THz spectroscopy on MEG. (**a**) Schematic diagram of the ultrafast time-resolved THz spectroscopy set-up. (Inset: schematic diagram of a MEG sample with a gradient doping density profile). (**b**) Normalized differential THz transmission spectra Δ*t*(*ω*)/*t*(*ω*) recorded at a pump fluence of 0.87 μJ cm^−2^ and a substrate temperature of 40 K for a few different pump–probe delays for a MEG sample with ∼63 layers. The black dashed line indicates the experimental noise level. The THz spectra are remarkably dispersionless in the detectable frequency range under all experimental conditions. (**c**,**d**) Linear (**c**) and logarithmic (**d**) plots of normalized differential THz transmission at the peak of the THz probe pulse Δ*t*/*t* as a function of pump–probe delay recorded at a pump fluence of 23.4 μJ cm^−2^ for a few different substrate temperatures for a MEG sample with ∼63 layers. The THz carrier dynamics evolve from a faster mono-exponential relaxation at room temperature to a slower bi-exponential relaxation at cryogenic temperatures. Subfigures (**c**) and (**d**) share the same legend. (**e**,**f**) Short and long relaxation times as a function of substrate temperature for a few different pump fluences for a MEG sample with ∼63 (**e**) and ∼35 (**f**) layers. The values are extracted from phenomenological fits to normalized differential THz transmission Δ*t*/*t*. The long relaxation times increase with the number of epitaxial graphene layers, which indicate the presence of interlayer interaction in MEG with HD and LD layers. (**g**) Normalized differential THz transmission at the peak of the THz probe pulse Δ*t*/*t* as a function of pump–probe delay recorded at a pump fluence of 60.0 μJ cm^−2^ for a few different substrate temperatures for a MEG sample with ∼3 layers. The THz carrier dynamics follow a fast mono-exponential relaxation at all temperatures. (**h**) Relaxation times as a function of substrate temperature for a few different pump fluences for a MEG sample with ∼3 layers. The relaxation times are completely independent of the substrate temperature, because there is practically very little or no interlayer energy transfer in MEG with all HD layers. The experimental error in all relaxation times is due primarily to long-term drift of the optomechanical components and the ultrafast Ti:sapphire laser system, and is estimated not to exceed ∼5%.

In the main body of this paper, we investigate the physics of interlayer Coulomb coupling in MEG, grown on the C-face of single-crystal 4H-SiC(
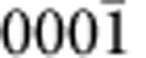
) substrates by thermal decomposition of Si atoms^1^,^2^. An important feature of this material is that it largely preserves distinct graphene-like electronic properties because of unique rotational stacking, which suppresses hybridization between low-energy electronic states localized in neighbouring planes of carbon atoms^7^,^8^. MEG is doped by electron transfer from the interface with the supporting SiC substrate, and the induced n-type carrier-density profile falls off rapidly with layer moving away from the substrate (see the inset of Fig. 2a). We will refer to the few layers close to the SiC substrate, which have large carrier densities of *n*_HD_≳10^12^ cm^−2^ as determined from high-resolution angle-resolved photoemission spectroscopy, scanning tunnelling spectroscopy, electronic transport and ultrafast optical spectroscopy measurements^1^,^2^,^15^,^16^,^20^, as HD layers, and to those further away, whose carrier densities drop quickly^21^ to the range of *n*_LD_≲10^10^ cm^−2^ as determined from scanning tunnelling spectroscopy, electronic transport and magneto-optical spectroscopy measurements^1^,^22^, as LD layers. The formation of local spatial charge inhomogeneities due to small amounts of disorder, impurities or surface corrugation of the SiC substrate could explain the non-zero carrier density measured in the top layers of MEG^23^,^24^,^25^.

### Electronic cooling in MEG

When a MEG sample is illuminated with a short optical pulse, electrons are excited to high energies, leaving behind unoccupied states or holes. Due to strong intralayer carrier–carrier scattering, these hot carriers thermalize with the background of cold carriers within ∼50 fs (refs 26, 27, 28, 29, 30), forming two separate non-equilibrium Fermi–Dirac distributions for electrons and for holes. The electron and the hole quasi-Fermi levels subsequently merge within ∼100–200 fs (refs 29, 30), establishing a uniform electron liquid within each layer *i* characterized by an elevated electron temperature, *T*_*i*_. As the electron liquid cools, each layer's electron temperature approaches the equilibrium lattice temperature, *T*_L_. It is generally accepted that initial fast cooling occurs in the first few picoseconds via the emission of energetic optical phonons, and that this process becomes increasingly inefficient as the electron energy falls below the relatively high optical phonon energy (*ħω*_op_≈200 meV (ref. 31)). In the final stage of relaxation, low-energy electronic cooling in an isolated layer would proceed by the much slower emission of acoustic phonons. Recent findings suggest that the low-energy phonon spectra of multilayer graphene systems are sensitive to the pattern of relative orientations^32^. Although this property will transfer a corresponding sensitivity to phonon cooling powers, relative orientations will not influence the interlayer Coulombic energy transfer mechanism explored here. Prior theoretical work has found that the rate of acoustic phonon cooling in disorder-free single-layer graphene is very strongly dependent on the carrier density, with cooling times ranging from tens of picoseconds for doping densities of ∼10^13^ cm^−2^ to tens of nanoseconds for doping densities of ∼10^10^ cm^−2^ (refs 10, 11). The strong carrier density dependence of the acoustic phonon emission implies that the LD and HD layers of MEG will exhibit very differing cooling rates following ultrafast optical excitation, leading to the buildup of a thermal gradient, which triggers an interlayer energy transfer.

We have applied two different experimental techniques to probe the dynamics of the interlayer energy transfer in MEG. The first is ultrafast time-resolved THz spectroscopy, which is a powerful tool for investigating the real-time relaxation dynamics of photoexcited carriers, because it is sensitive to both the number of carriers and their distribution in energy^33^,^34^. Due to the large number of layers in our MEG samples and the rapid decrease of carrier density with layer number, the measured differential THz transmission signal is dominated by the dynamic THz response of the many LD layers and reveals the cooling of the LD layers due to their coupling to the HD layers. The second is ultrafast degenerate IR pump–probe spectroscopy, in which we optically inject hot carriers selectively into the LD layers and then observe directly the transfer of heat to the electron liquid in the most highly doped HD layer^15^,^16^.

### Ultrafast time-resolved THz spectroscopy

We first consider the ultrafast time-resolved THz spectroscopy experiments. We report measurements on a series of MEG samples ranging from ∼3 to ∼63 layers in an ultrafast optical-pump THz probe set-up^33^,^34^ as illustrated schematically in Fig. 2a. Our laser system consists of a Ti:sapphire oscillator (Mira 900-F, Coherent) followed by a Ti:sapphire regenerative amplifier (RegA 9050, Coherent) and produces ultrafast optical pulses with a centre wavelength of 800 nm, a pulse width of ∼60 fs and a repetition rate of 250 kHz. A portion of the laser beam is quasi-collimated at the sample position with an intensity spot size diameter of ∼1,600 μm, and optically injects hot carriers in the MEG samples. A second portion of the laser beam is used to generate a single-cycle THz pulse in a low-temperature-grown GaAs photoconductive emitter (Tera-SED 3/4, Gigaoptics)^35^,^36^, and the emitted broadband THz radiation is focused on the MEG sample with an intensity spot size diameter of ∼500 μm to probe the dynamic THz response. The transmitted portion of the THz probe is detected using time-domain electro-optic sampling in a 1-mm-thick ZnTe crystal^37^,^38^,^39^ and a pair of balanced Si photodiodes. The electrical signal is modulated by a mechanical chopper, placed in either the optical pump or the THz probe arm, and recorded using a conventional lock-in amplifier data acquisition technique. The MEG sample is mounted inside a liquid helium continuous flow cryostat (ST-100, Janis), so that the substrate temperature can be varied from 10 to 300 K. The time delays between the optical pump, the THz probe and the sampling pulse are controlled by two motorized stages. All THz optics is surrounded by an enclosure purged with purified nitrogen gas to minimize water vapour absorption. The detection bandwidth of the system is in the range of ∼0.2–2.5 THz, and the temporal resolution of the measurements is limited by the duration of the THz probe pulse to the sub-picosecond timescale. The experimental error is due primarily to the long-term drift of the optomechanical components and the ultrafast Ti:sapphire laser system, and is estimated not to exceed ∼5%.

As a first experimental approach, we measure the differential change in the THz probe pulse transmission through the MEG sample due to photoexcitation. The THz probe field is recorded in the time domain, and it is later numerically Fourier transformed to obtain the frequency spectrum. Figure 2b shows the differential THz transmission spectra normalized to the THz transmission without photoexcitation, Δ*t*(*ω*)/*t*(*ω*), for a few different THz probe delays after the optical pump for a MEG sample with ∼63 layers. From the Tinkham formula for the transmission through a thin conducting film on a transparent substrate^40^, the Δ*t*(*ω*)/*t*(*ω*) signal can be directly related to the photoinduced change in the complex sheet conductivity of MEG, Δ*σ*(*ω*), through the expression:


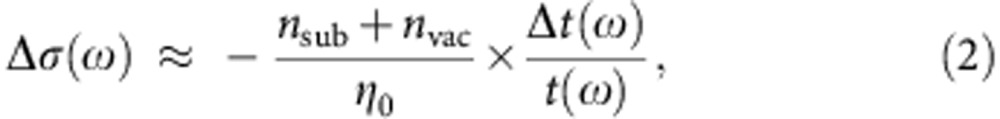


where *n*_sub_ and *n*_vac_ are the THz refractive indices of the SiC substrate and the environment, respectively, and *η*_0_ is the impedance of free space. The THz conductivity of MEG is a summation of the THz conductivities of the individual epitaxial graphene layers in the MEG stack, because the layers are electronically decoupled^7^,^8^. Additional THz transmission spectra, similar to the ones in Fig. 2b, but for variable substrate temperature and variable pump fluence, are shown in Supplementary Figs 1 and 2; Supplementary Note 1. We note that the normalized differential THz transmission spectra are remarkably dispersionless in the detectable frequency range under all experimental conditions; this justifies the application of a simpler data acquisition scheme in which we record the normalized differential THz transmission only at the peak of the THz probe pulse.

As a second experimental approach, we keep the delay of the sampling pulse fixed at the peak of the THz probe pulse, and we scan the pump–probe delay to map out the relaxation dynamics of the photoexcited carriers. Since the carrier–carrier scattering time in graphene is much shorter than the temporal duration of the THz probe, we study the relaxation of the THz transmission (or the THz conductivity) change induced by the optical pump in the limit, where it is determined by collective electronic cooling dynamics. Figure 2c,d shows the normalized differential THz transmission at the peak of the THz probe pulse, Δ*t*/*t*, as a function of pump–probe delay for variable substrate temperature for the same MEG sample with ∼63 layers. At time zero, the optical pump photoexcites carriers in the MEG sample resulting in an overall increase of the THz conductivity and hence THz absorption, as manifested in a negative differential THz transmission. In graphene with very low doping, the increase of the electron temperature leads primarily to larger electron occupation in the conduction band and a corresponding net increase of the THz conductivity, consistent with our interpretation that the measured dynamic THz response is dominated by the hot carriers in the many LD layers of MEG^41^,^42^,^43^. The differential THz transmission reaches its maximum magnitude within ∼1 ps, with the rise time being limited mainly by the temporal duration of the THz probe. The differential THz transmission subsequently recovers as the thermalized hot carriers cool to the substrate temperature with relaxation times ranging from a few picoseconds at room temperature to hundreds of picoseconds at cryogenic temperatures. The secondary decrease in the differential THz transmission at ∼7 ps is due to a round-trip reflection of the optical pump inside the substrate that photoexcites additional carriers.

We perform phenomenological fits to the experimental data in Fig. 2c,d, and we discover that the differential THz transmission evolves from a faster mono-exponential decay at room temperature to a slower bi-exponential decay at cryogenic temperatures. As we explain below, the slow electronic cooling at low substrate temperatures is controlled by interlayer Coulombic energy transfer between the LD and HD layers. A summary of the extracted carrier relaxation times as a function of substrate temperature for a few different pump fluences is presented in Fig. 2e. We observe that the relaxation times are largely independent of the pump fluence (or the initial electron temperature), except at high substrate temperatures. The slight increase in the relaxation times at the highest pump fluence can be attributed to heating of the HD layers above the substrate temperature, which decreases the rate of the energy transfer from the LD layers. Similarly, the energy transfer between layers becomes less efficient at high substrate temperatures, at which the difference between the electron temperatures in different layers is small. As a consequence, the contribution of the interlayer Coulomb coupling to electronic cooling is diminished at substrate temperatures above ∼200 K, as evidenced from the fits.

We repeat identical experiments and analysis for a second MEG sample with ∼35 layers, and the corresponding summary of the extracted carrier relaxation times for the best fits are presented in Fig. 2f. Qualitatively, the THz carrier dynamics for the 35-layer sample mirror those for the 63-layer sample by exhibiting a transition from a faster mono-exponential to a slower bi-exponential decay as the substrate temperature is decreased. Similarly, the relaxation times are independent of the pump fluence, except at high substrate temperatures. Further inspection shows that the long relaxation times become up to a few times shorter when the number of epitaxial graphene layers is nearly halved, which indicates the presence of interlayer interaction. We show below that because of the range dependence of the Coulomb scattering processes, the addition of more LD layers slows their collective electronic cooling via coupling to the HD layers.

To underscore the profound influence of interlayer energy transfer on the electronic cooling in MEG, we next study the limiting case of MEG with all HD layers. We again perform identical experiments and analysis for a third MEG sample with only ∼3 layers. Figure 2g shows the normalized differential THz transmission at the peak of the THz probe pulse, Δ*t*/*t*, as a function of pump–probe delay for variable substrate temperature. First, we note that the differential THz transmission for MEG with all HD layers is positive, which has been previously phenomenologically attributed to enhanced carrier scattering as the electron temperature is elevated^42^,^43^. Second, we observe that the THz carrier dynamics are much faster and completely independent of the substrate temperature, because there is practically very little or no interlayer energy transfer. The differential THz transmission is fit very well by a phenomenological mono-exponential decay at all temperatures (again accounting for the substrate reflection of the optical pump), and the summary of the extracted carrier relaxation times as a function of substrate temperature for a few different pump fluences is presented in Fig. 2h. These relaxation dynamics are inconsistent with the disorder-assisted electron–phonon (supercollision) cooling mechanism (see Supplementary Figs 5–10; Supplementary Note 6), but they could be attributed to the optical and acoustic phonon cooling mechanisms of hot carriers.

### Ultrafast degenerate IR pump–probe spectroscopy

To further support the existence of interlayer energy transfer in MEG, we devise another experiment using ultrafast degenerate IR pump–probe spectroscopy^15^,^16^ in which we selectively photoexcite hot electrons in all layers of MEG except the first HD layer nearest to the SiC substrate. We then observe the cooling of these hot electrons via interlayer Coulomb coupling to the cold electrons in the first HD layer. Our laser system consists again of a Ti:sapphire oscillator and amplifier followed by an optical parametric amplifier (OPA 9850, Coherent) and produces ultrafast optical pulses with a centre wavelength tuned to 1.8 μm. The OPA beam is filtered through a 10-nm-bandpass filter centred at 1.8 μm, and is split into a pump and a probe beams that are both focused on the MEG sample with an intensity spot size diameter of ∼50 μm. The transmitted portion of the probe is detected using a grating spectrometer and an InGaAs photodetector in conjunction with a conventional lock-in amplifier data acquisition technique. The MEG sample is mounted inside a cryostat (ST-100, Janis), and the substrate temperature is held at 10 K. The experimental error is again estimated not to exceed ∼5%.

**Figure 3:**
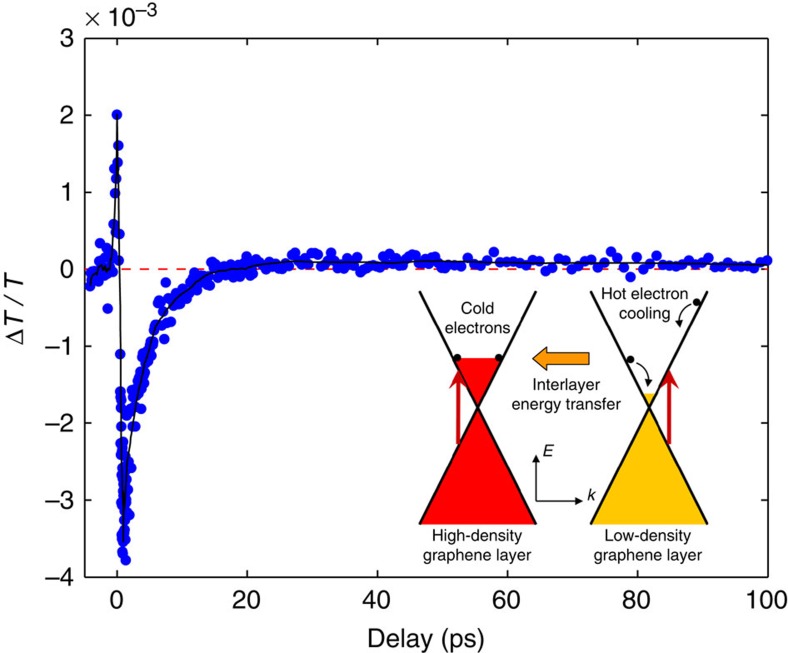
Ultrafast degenerate IR pump–probe spectroscopy on MEG. Normalized probe differential transmission Δ*T*/*T* in ultrafast degenerate 1.8-μm IR pump–probe spectroscopy as a function of pump–probe delay recorded at a pump fluence of 80 μJ cm^−2^ and a substrate temperature of 10 K for a MEG sample with ∼63 layers. The black solid line is a guide for the eye. The sign changes in the differential transmission at ∼1 and ∼20 ps indicate the presence of interlayer energy transfer from the top layers to the first HD layer in MEG. (Inset: schematic diagram of interlayer Coulombic energy transfer from a hot LD to a cold HD layer in MEG. The pump selectively injects hot electrons in all layers of MEG except the first HD layer, in which interband absorption is Pauli blocked).

In this experimental approach, both the pump and the probe photon energies (*ħω*≈690 meV) are chosen to be slightly smaller than twice the Fermi level of the first HD layer (*E*_F_≈360 meV), but larger than twice the Fermi levels of all other layers in MEG^15^,^16^. Thus, the pump selectively injects hot electrons in all layers of MEG except the first HD layer, in which interband absorption is Pauli blocked as illustrated schematically in the inset of Fig. 3. The probe differential transmission due to photoexcitation has a positive contribution from the hot electrons in all layers of MEG, except the first HD layer. The first HD layer gives no contribution, when the carriers in that layer remain unexcited. However, interlayer Coulombic energy transfer can heat this layer, which results in a distinct negative contribution to the differential transmission.

Figure 3 shows the probe differential transmission normalized to the probe transmission without photoexcitation, Δ*T*/*T*, as a function of pump–probe delay for the MEG sample with ∼63 layers. We observe that immediately after photoexcitation the differential transmission is positive, arising from the hot electrons that are directly injected in the top layers. Shortly after that, the differential transmission becomes negative and reaches its minimum value within ∼1 ps. This sign change demonstrates the existence of an efficient interlayer energy transfer, in which the cold electrons in the first HD layer act as a heat sink for the hot electrons in the top layers. The rapidly rising electron temperature in the first HD layer has a dominant negative contribution to the differential transmission. As delay time increases, the electrons in the first HD layer cool much faster than the electrons in the top layers due to the sharply increasing rate of acoustic phonon emission with carrier density. This results in a second sign change in the differential transmission at ∼20 ps, when the electron temperature in the first HD layer approaches the equilibrium lattice temperature. By comparing with the THz carrier dynamics in Fig. 2g, we note that it takes slightly longer for the HD layer to cool due to the extra heat from the top LD layers. At that point, the optical and acoustic phonon cooling rate in the first HD layer balances the interlayer energy transfer rate from the top layers. Electronic cooling in the top layers of MEG via the interlayer Coulomb coupling survives on a timescale exceeding a hundred picoseconds (limited by the experimental signal-to-noise ratio), which is consistent with that observed in the THz carrier dynamics in Fig. 2c,d. Now, we turn our attention to a detailed presentation of the theory of hot-carrier equilibration based on interlayer energy transfer via screened Coulomb interactions.

### Interlayer Coulombic energy transfer theory

Non-equilibrium electrons in graphene have been shown to thermalize within a layer on an ultrafast timescale on the order of tens of femtoseconds^26^,^27^,^28^,^29^,^30^. We assume that this property holds also in MEG, and that it leads to pseudo-equilibrium electronic states with well-defined temperatures *T*_*i*_ in layer *i*, but we allow for the possibility of differences in temperature between layers that survive to longer timescales. The multilayer temperature dynamics are described by a set of coupled nonlinear first-order differential equations:


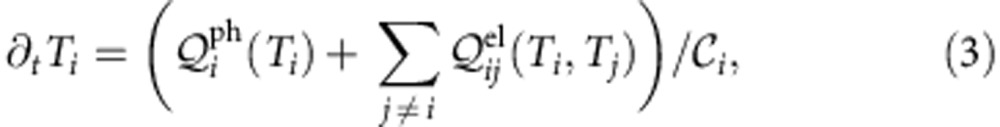


where 
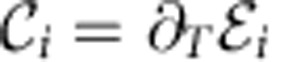
 is the heat capacity and 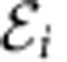 the energy density of electrons in the *i'*th layer. The rate of change of energy density in layer *i* is determined by the sum of two processes: energy loss to the lattice via electron–phonon scattering at rate 
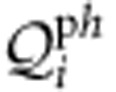
, and energy transfer from other layers *j* to *i* via interlayer electron–electron scattering at rate 
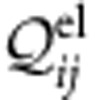
. Both of these mechanisms depend strongly on the carrier density. The four most highly doped layers in the 63-layer MEG sample have Fermi energies measured to be 360, 218, 140 and 93 meV, respectively^16^. A simple Thomas–Fermi model^44^ is able to account semi-quantitatively for the monotonic decrease in carrier density with separation from the substrate in multilayer graphene systems. Electronic transport and magneto-optical spectroscopy measurements^1^,^22^ of top LD layers suggest local carrier density fluctuations in these LD layers that satisfy *n*_LD_≲10^10^ cm^−2^.

Recent ultrafast optical spectroscopy experiments^15^ have found that the hot carriers in the HD layers of MEG quickly relax to the lattice temperature with equilibration times on the order of a few picoseconds, in good agreement with the experiments reported here. Theoretical calculations neglecting interlayer thermal coupling (
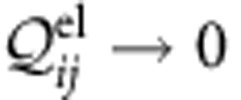
) have obtained order of magnitude agreement with these measurements^10^. On the other hand, the same approximation applied to the LD layers erroneously predicts thermal equilibration times on the order of several nanoseconds, in sharp disagreement with the experiments reported here. This was the initial impetus for our examination of the interlayer Coulombic energy transfer mechanism. According to ref. 10, the acoustic phonon cooling power is proportional to the square of the carrier density, *n*^2^. Allowing for disorder-assisted electron–phonon (supercollision) scattering changes this dependence to *n* (ref. 14). In the HD layers, optical and acoustic phonon emission is likely to provide the dominant cooling pathway for hot carriers^15^. In the LD layers, however, we suggest that it plays a more subsidiary role by keeping the electron temperature in the HD layers pinned to the lattice temperature (*T*_HD_=*T*_L_), while they act as a Coulomb-coupled heat sink for the LD layers (see Supplementary Fig. 4; Supplementary Note 5).

Although the true electron density profile across the many layers of MEG is expected to decrease smoothly, it is convenient to make a sharp distinction between highly doped (HD) and lightly doped (LD) layers. We predict that the asymptotic temperature dynamics of LD layers in MEG are effectively governed by equation (3) with 
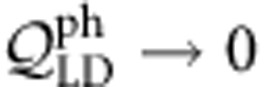
. In our theoretical analysis, we will denote the four most highly doped layers near the substrate as HD layers. Quantitatively, this cutoff is suggested from the disorder-free theory of acoustic phonon cooling^10^, which, in combination with equation (4) in Supplementary Note 2, implies that the hot-electron distribution in layers *i*>4 transfers energy via the interlayer Coulomb interaction to the *j* HD layers (*j*<*i*) faster than it loses energy to the lattice via acoustic phonon emission. More precisely, we find that 
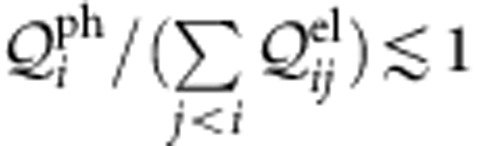
, for temperatures *T*_*i*_≳50 K. In all calculations described below, we use the values *E*_F,1–4_=360, 218, 140 and 93 meV for the Fermi levels of the first four HD layers. These have been measured explicitly for the 63-layer MEG sample and are expected to be good estimates for the 35-layer MEG sample.

The rate of Coulombic energy transfer between two layers is given by a Fermi golden-rule expression:


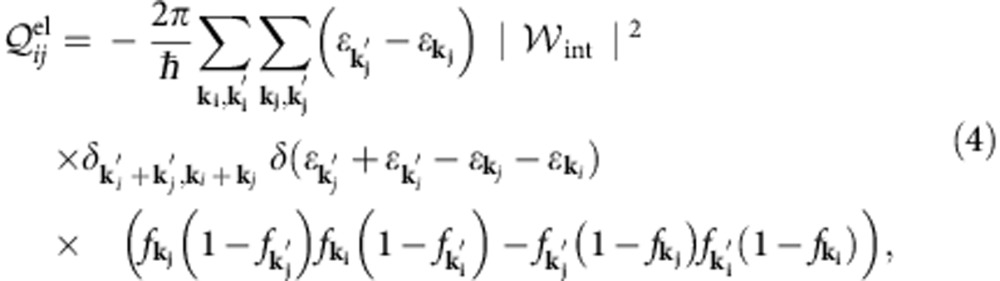


where (*i*, *j*) are layer indices, and **k** is a collective index that implies, in addition to wavevector, the spin, the valley and the band index labels required to specify single-electron states in graphene's low-energy Dirac model^45^. As mentioned above, intralayer electron thermalization^26^,^27^,^28^,^29^,^30^ is much faster than interlayer energy transfer, and this allows us to describe electronic state occupations with a quasi-equilibrium Fermi distribution *f*_**k**_=(*ɛ*_**k**_,*μ*,*T*). In the random-phase approximation (RPA):


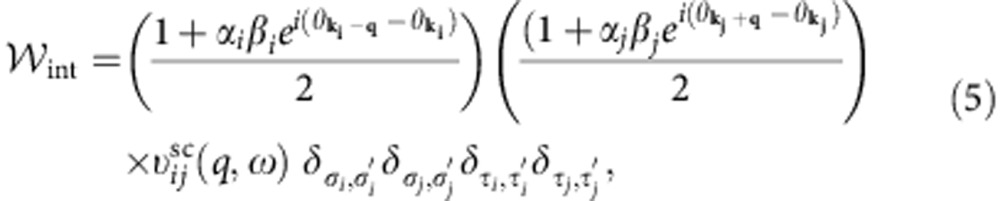


where *q*=|**k**_*j*_′–**k**_*j*_|, 
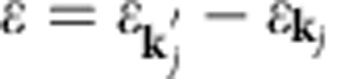
 and 
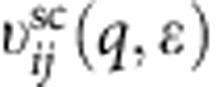
 is the screened electron–electron interaction between layers *i* and *j* (see below). When screening is neglected, 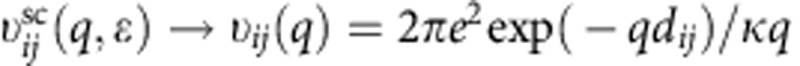 is the 2D Fourier transform of the bare Coulomb interaction between two electrons with interlayer separation *d*_*ij*_ and *κ*=5.5 to account for the presence of MEG at the surface of the SiC substrate. The factors in parenthesis are the well-known form factors that account for the sub-lattice spinor dependence of graphene π-band plane-wave matrix elements. The indices *α* and *β* are equal to 1 and −1 for conduction and valence band states, respectively. The Kronecker delta's in equation (5) explicitly exhibit the property that continuum model interactions are independent of spin (*σ*) and valley (*τ*). Using equation (5) and comparing with the expression for graphene's non-interacting density–density response function, *χ*(*q*,*ω*,*T*), we finally obtain the following compact expression which is suitable for numerical evaluation:


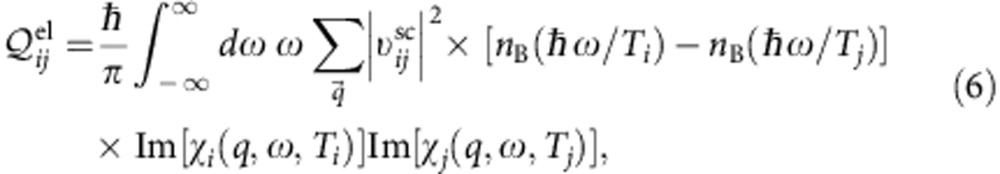


where *n*_B_(*x*)=1/(exp(*x*)−1) and *k*_B_=1 throughout. We use this expression below to calculate interlayer energy transfer rates. A central quantity in the theoretical formulation of the many-body effects of Dirac fermions is the dynamical polarizability tensor *χ*_*i*_(*q*,*ω*,*T*_*i*_) for the *i*'th layer at temperature *T*_*i*_. This is defined through the one-body non-interacting Green's functions^46^. The density–density response function of the doped 2D Dirac electron model was first considered by Shung^47^ at zero temperature as a step towards a theory of collective excitations in graphite. The Dirac electron expression *χ*_*i*_(*q*,*ω*,*T*_*i*_) at finite temperature has been recently considered^48^,^49^,^50^. Before proceeding with the rate calculations, however, we must first discuss the approximation we use for the screened interlayer Coulomb interaction.

In the RPA, the bare interlayer Coulomb interaction is screened^51^ by the potential produced by self-consistently readjusted charge density. For a general multilayer system, this implies the following^52^:


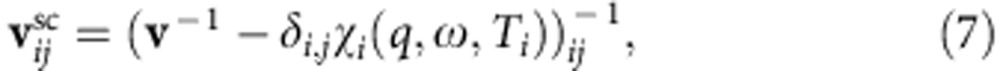


where **v** and **v**^sc^ are matrices that describe bare and screened potentials, respectively, in one layer (*i*) to an external charge in another layer (*j*). Screening complicates energy cooling dynamics, because it causes the energy transfer rate between a particular pair of layers to depend, through *χ*_*i*_(*q*,*ω*,*T*_*i*_), on the temperatures in all layers. However, the low carrier densities in the layers far from the substrate^1^,^2^ motivate a simplifying approximation in which their contributions to screening are neglected. By comparing this approximation with the full expression, we find that this simplification is justified in the regions of phase space (*q*,*ω*) important for low-temperature energy transfer.

The relative ability of intraband excitations in LD and HD layers to screen the interaction can be established by examining the ratio of their density response functions in the static limit: 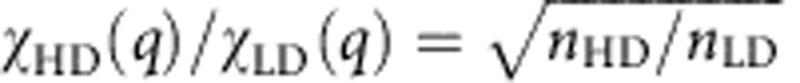. In our MEG samples this ratio varies within ∼8–31 in the important regions, and suggests that the leading order charge polarization responsible for screening the MEG interlayer Coulomb interaction can be approximated without including the contribution from the LD layers. We note that the Bose factors in equation (6) limit transfer energies to *ħω*≲*k*_B_*T*. We also note that the factor exp(−*qd*_*ij*_) in the interlayer Coulomb interaction limits the important wavevector transfers to *q*≲1/*d*_*ij*_. The conditions (setting *ħ* and *k*_B_→1),


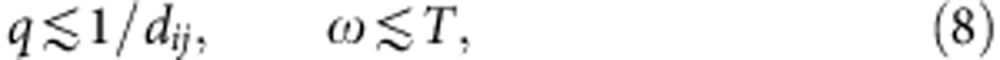


apply equally well to Coulomb-mediated interlayer energy and momentum transfer in any type of multilayer 2D electron system.

Setting *χ*_*j*_(*q*,*ω*,*T*_*j*_) to zero for all but the four HD layers nearest the substrate, and letting the separation distance between the four HD layers also go to zero (relative to the generally much larger spacing between HD and LD layers in MEG), we obtain the following approximate expression:


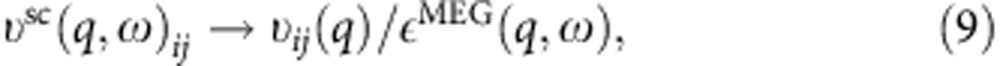


where


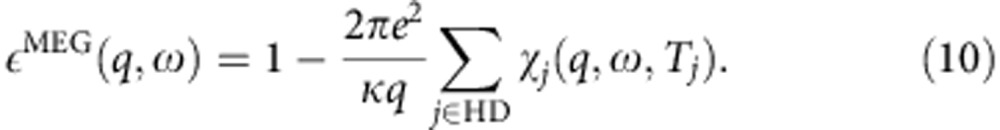


In the calculations reported on below index *j* was summed over the four HD layers.

Since cooling powers between LD layers are typically one to two orders of magnitude larger than between LD and HD layers^53^ (
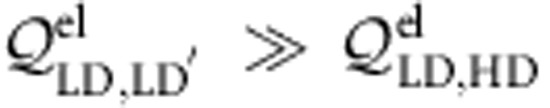
), energy transfer between pairs of LD layers cannot be ignored in the calculations. Poles in the screened interaction described by equation (7) at plasmon frequencies greatly enhance the interlayer quasi-particle scattering rate. This effect does not contribute to energy transfer between HD and LD layers, because the plasmon modes then lie at higher frequencies than can be excited in low-temperature HD layers; the plasmon poles reside above the intraband particle–hole continuum of HD layers, that is, 
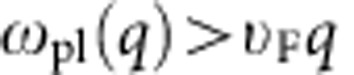
. However, at temperatures comparable to the Fermi temperature, interband particle–hole excitations are no longer Pauli blocked at any frequency, and these excitations can take advantage of the plasmon poles in the screened interaction^54^,^55^. It is the small Fermi temperatures (
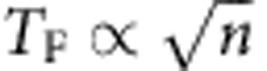
) of the LD layers that markedly increase the Coulomb coupling among the group of LD layers.

**Figure 4:**
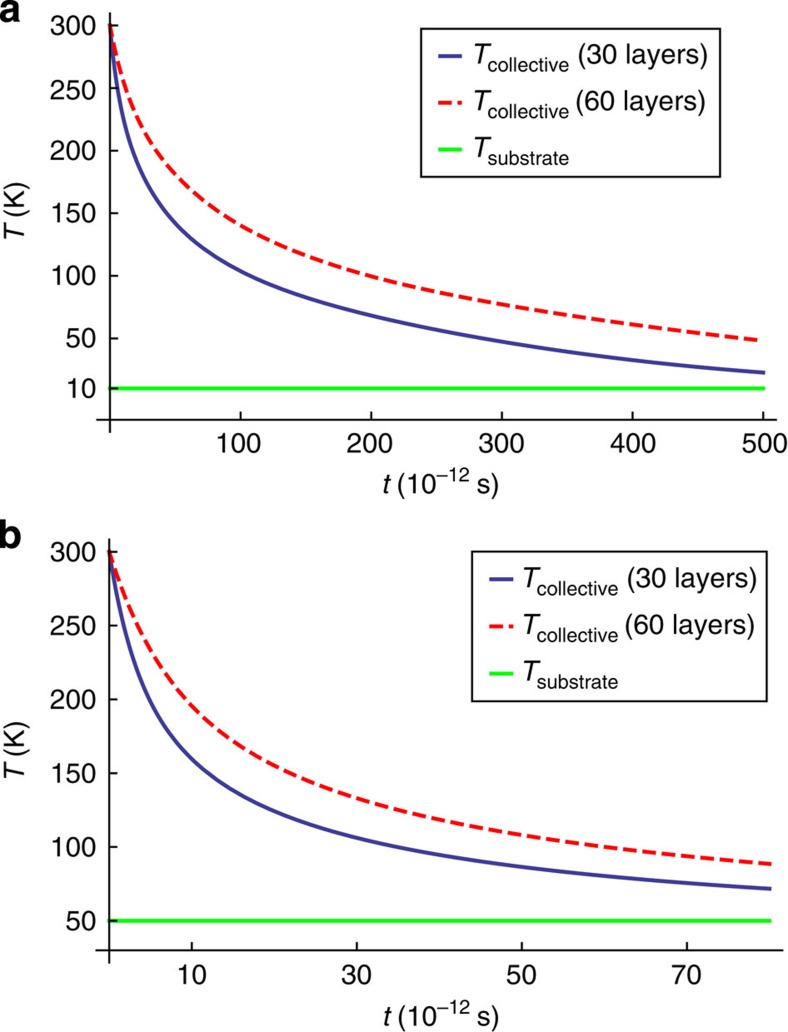
Interlayer Coulombic energy transfer in MEG. Collective cooling dynamics of the LD layers in MEG with 30 and 60 layers, calculated using equation (11) for lattice temperature *T*_L_=10 K (**a**) and *T*_L_=50 K (**b**). Both figures illustrate that the MEG hot-carrier relaxation characteristics observed in Fig. 2 are naturally described by the interlayer Coulombic energy transfer mechanism. MEG samples with more LD layers cool more slowly because Coulomb coupling to the HD layers close to the substrate decreases with increasing layer separation.

As LD layers closer to the substrate cool, they absorb energy from and cool more distant LD layers. This effect results in a collective cooling state in which the electron temperatures of all LD layers relax to the equilibrium lattice temperature nearly uniformly, and motivates an approximation with a single collective LD layer temperature, *T*_c_(*t*). In employing this approximation, our goal is to establish that Coulomb scattering is a relevant energy transfer process up to quite high temperatures. A more detailed calculation in which the temperature of each LD layer is allowed to vary independently would be warranted if the charge density profile in the multilayer system was accurately known. Such a calculation might in any event not achieve greater accuracy since the calculations of electron–electron scattering amplitudes can only be performed approximately. The collective cooling state model we employ has the advantage that it requires fewer computationally burdensome finite-temperature dynamic polarizability calculations. The collective cooling dynamics model of the collective temperature is given by:


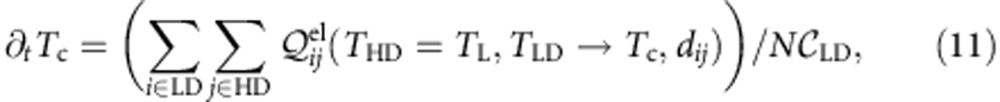


where *N* is the total number of LD layers. We have summed over the energy transfer rate between all LD–HD pairs of layers, appealing to strong electron–phonon coupling to keep the HD layers temperature at *T*_L_ and strong 
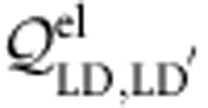
 to keep the LD layers at a common temperature *T*_c_(*t*). Figure 4a,b compares the calculated collective thermal relaxation dynamics *T*_c_(*t*) in 30-layer and 60-layer MEG samples for lattice temperature of 10 and 50 K. For both lattice temperatures, thicker MEG samples cool more slowly. This trend can be understood by noting that each additional LD layer contributes an equal amount to the combined LD layers heat capacity, whereas the energy transfer rate to the HD layers falls off upon moving further away from the substrate (see Supplementary Fig. 3; Supplementary Note 4).

If we linearize the collective temperature of the LD layers about the equilibrium lattice temperature, δ*T*≡*T*_c_(*t*)–*T*_L_, we find that the interlayer energy transfer rate can be written as follows:


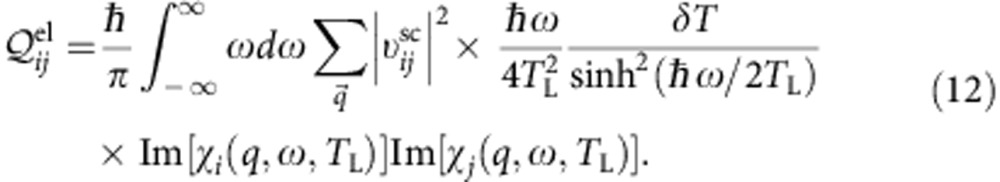


**Figure 5:**
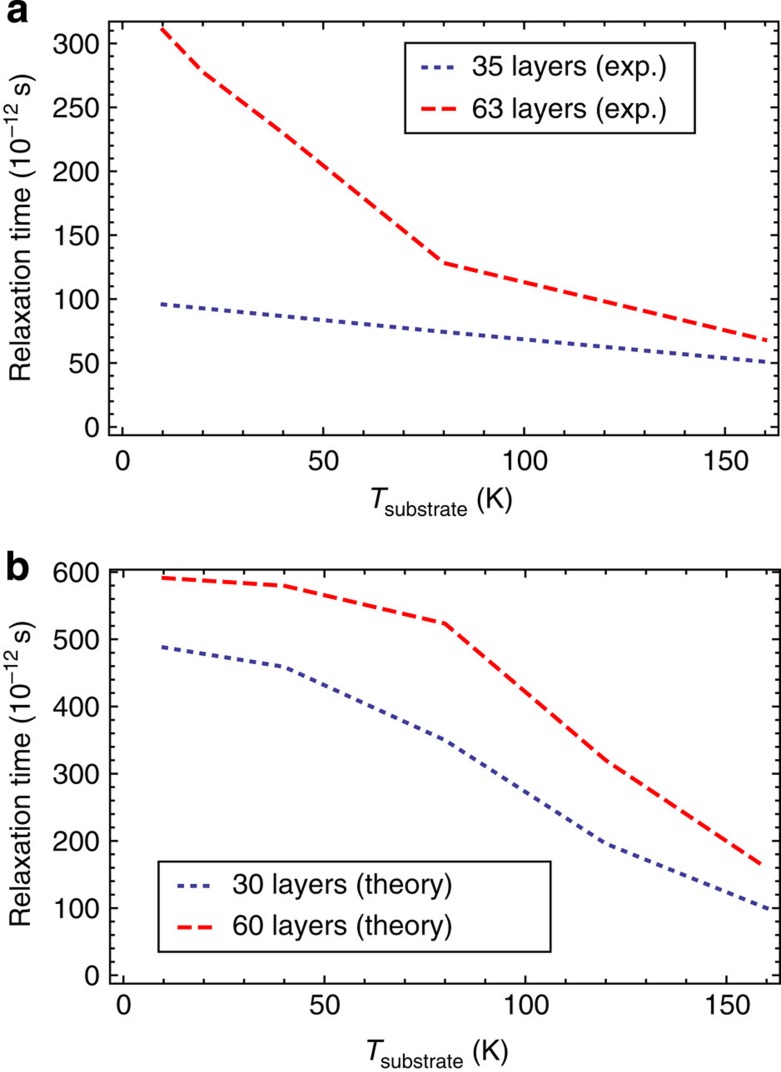
Comparison between experimental and theoretical relaxation times in MEG. (**a**) Experimental hot-carrier relaxation times versus substrate temperature for MEG samples with ∼63 and ∼35 layers. The values correspond to the long relaxation times from Fig. 2e,f. (**b**) Theoretical hot-carrier relaxation times versus substrate temperature calculated using the linearized interlayer Coulombic energy transfer rate equation (12). The interlayer Coulombic energy transfer mechanism explains relaxation time trends versus substrate temperature and multilayer system thickness. The factor-of-two discrepancy in magnitude is typical for RPA theories of electron-electron scattering amplitudes. The level of agreement found here is similar to that found in comparisons of RPA calculations and measurements of the Coulomb-coupled interlayer momentum transfer rate (that is, Coulomb drag resistivity) between neighbouring GaAs/AlGaAs quantum wells^54^.

This equation can be used to define collective electronic cooling times that are easier to compare with the experimental carrier relaxation times extracted from the phenomenological fits. Figure 5 compares the experimental and the theoretical relaxation times over a lattice temperature range of *T*_L_=10–160 K. From this figure we see that, within our approximations, theory reproduces the experimental trends versus both the lattice temperature and the number of epitaxial graphene layers; more specifically, both relaxation times increase with decreasing lattice temperature and increasing the number of layers. We note that since our theoretical relaxation times are obtained by linearizing the interlayer energy transfer rate equations, they underestimate interlayer coupling except when *T*_LD_–*T*_HD_<<*T*_HD_. Thus, we expect (and observe) the theoretical relaxation times to be longer than those obtained by fitting the experimental data over a broader temperature range.

We can identify the microscopic origins of the relaxation times' dependence on lattice temperature using a formula derived in Supplementary Note 2, where we give an analytic formula for the interlayer Coulombic energy transfer rate between a single LD layer and a single HD layer in MEG. Here, we show only the result of summing over the rates between all HD–LD pairs of layers. Applying this result to the multilayer case, we obtain the following net energy loss rate (per area) for *N* LD layers in MEG:


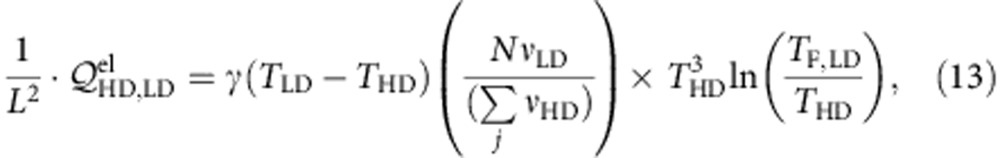


where 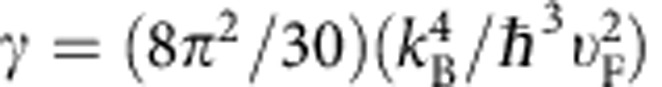 and the density of states in the HD and LD layers are denoted by 
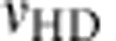
 and 
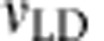
, respectively. The sum over index *j* runs over all HD layers in the MEG sample. We find that the interlayer Coulombic energy transfer rate exhibits a temperature dependence of *T*^3^ln(*T*) (ref. 56). This result is related to the familiar result^51^ for electron–electron scattering rates *τ*^−1^ in Fermi liquids, which are proportional to *T*^2^ln(*T*). These power laws appear, because the Fermi distribution limits the initial–final state pair energies that can partake in scattering to an energy window of width *ħω*∼*k*_B_*T* about the Fermi energy, which qualitatively explains the additional factor of *T*; the additional factor of *T* appears, because scattering events are weighted by the transferred energy in the cooling power case. On the other hand, the heat capacity of nearly neutral graphene increases linearly with *T* (or as *T*^2^ if *T*<<*T*_F_). The final result is that the electronic cooling time defined above becomes shorter with increasing lattice temperature, consistent with the experimental trends as shown in Fig. 5. Precise agreement is not expected outside of the degenerate temperature regime (*T*_L_<<*T*_F,LD_∼135 K), within which equation (13) becomes exact.

## Discussion

In conclusion, we have developed a theory of hot-carrier equilibration based on interlayer energy transfer via screened Coulomb interactions between electrons in the many top LD layers and in the few HD layers close to the underlying SiC substrate in MEG. The theory is complicated by the essential role of dynamic screening of the electron–electron interactions in all MEG layers through the temperature-dependent charge carrier response. To obtain a transparent theory, we have made two well-justified simplifications. First, we note that screening in the relevant temperature, wavevector and frequency regime is dominated by the first few HD layers close to the substrate, allowing us to neglect the screening by the top LD layers. Second, we note that interlayer energy transfer among the LD layers is much stronger than between LD and HD layers, and we therefore can describe all LD layers by a common electron temperature. We have compared the calculated cooling dynamics with the relaxation dynamics measured via ultrafast time-resolved THz spectroscopy. The observed experimental dynamics exhibit the expected timescales, dependence on lattice temperature and dependence on number of epitaxial graphene layers predicted by the theory, providing strong support for the proposed mechanism, within the approximations necessary in the development of the theory. The theoretical approach developed here may be expected to be applicable to many other types of layered 2D electron systems. These may include semiconductor heterostructures as well as the wide variety of novel 2D materials under active development including transition metal dichalcogenides (for example, MoS_2_, MoSe_2_, WS_2_, WSe_2_ and so on)^5^, and other van der Waals heterostructures^3^,^4^.

## Additional information

**How to cite this article:** Mihnev, M. T. *et al*. Electronic cooling via interlayer Coulomb coupling in multilayer epitaxial graphene. *Nat. Commun.* 6:8105 doi: 10.1038/ncomms9105 (2015).

## Supplementary Material

Supplementary Information Supplementary Figures 1-10, Supplementary Notes 1-6 and Supplementary References
