# Supplementary material for: Electronic cooling via interlayer Coulomb coupling in multilayer epitaxial graphene
Source: Nat Commun. 2015 Sep 24;6:8105. doi: 10.1038/ncomms9105 (PMC4598362; doi:10.1038/ncomms9105)
Supplement: Supplementary Information — Supplementary Figures 1-10, Supplementary Notes 1-6 and Supplementary References [file ncomms9105-s1.pdf]

SUPPLEMENTARY FIGURES

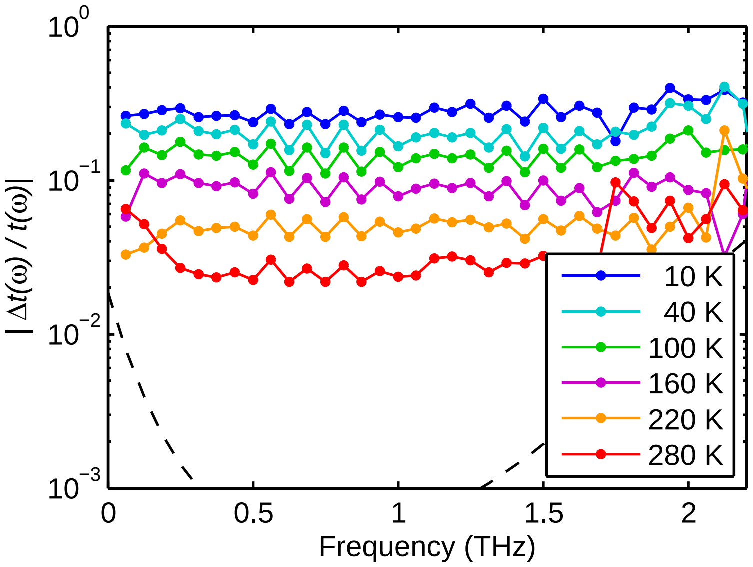

Supplementary Figure 1. **Ultrafast time-resolved THz spectroscopy on MEG.** Normalized differential THz transmission spectra $\Delta t(\omega)/t(\omega)$ recorded at a pump fluence of 0.87 μJ cm^{-2} and a pump-probe delay of 1 ps for a few different substrate temperatures for a MEG sample with ~ 63 layers. The black dashed line indicates the experimental noise level.

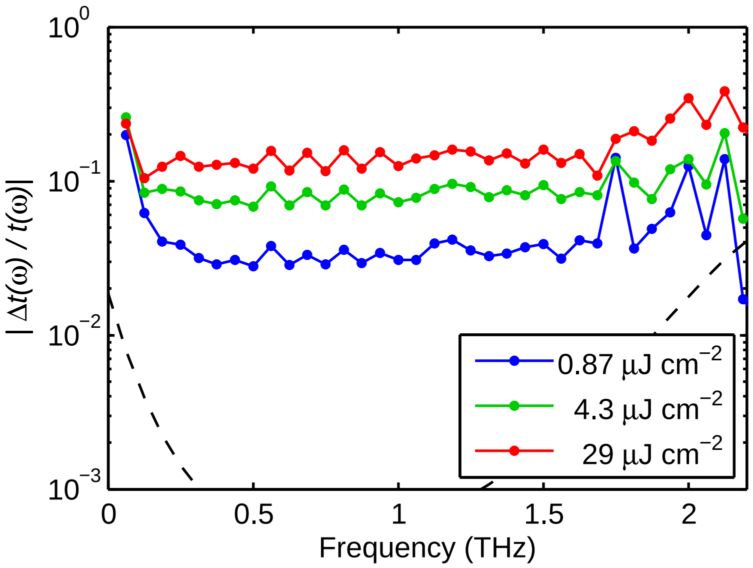

Supplementary Figure 2. **Ultrafast time-resolved THz spectroscopy on MEG.** Normalized differential THz transmission spectra $\Delta t(\omega)/t(\omega)$ recorded at a substrate temperature of 280 K and a pump-probe delay of 1 ps for a few different pump fluences for a MEG sample with ~ 63 layers. The black dashed line indicates the experimental noise level.

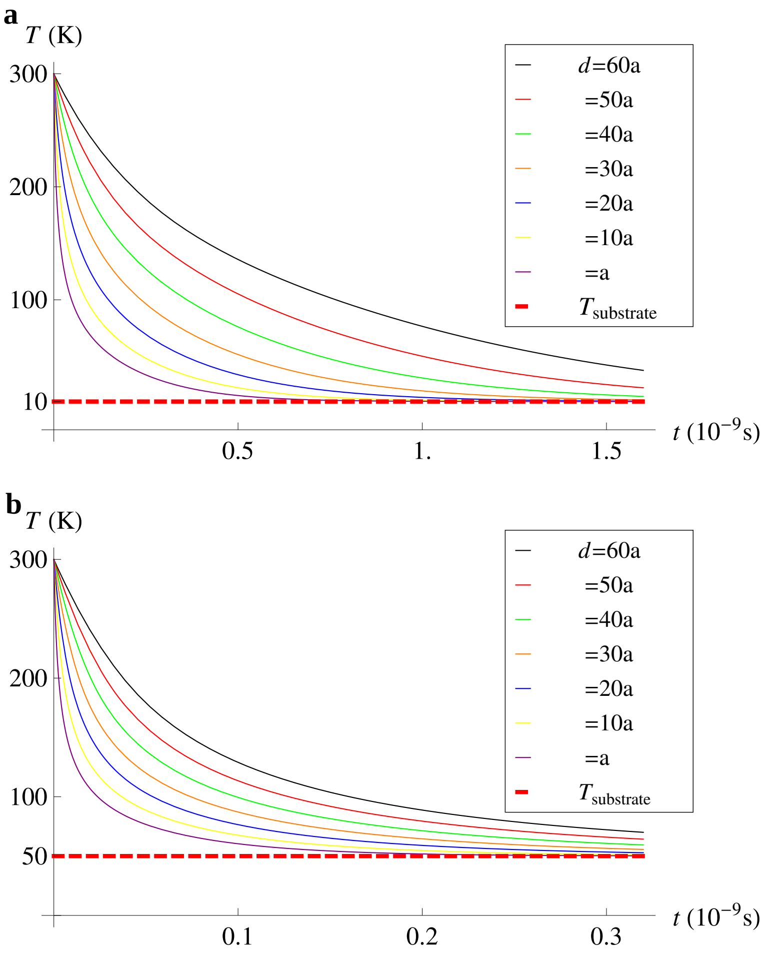

Supplementary Figure 3. **Interlayer energy transfer with no LD-LD layer coupling.** Temperature dynamics when interlayer energy transfer between LD layers ($n_{\mathrm{LD}} = 10^{10}\mathrm{cm}^{-2}$) is ignored. The LD layer temperature $T_{\mathrm{LD}}(t)$ resulting from cooling via interlayer Coulombic energy transfer to the HD layers near the substrate ($n_{\mathrm{HD}} \gtrsim 10^{12}\mathrm{cm}^{-2}$) at a constant lattice temperature T_{L}. The distance between the particular LD layer and the HD layers is varied, $d_{\mathrm{HD,LD}} = 60a, 50a, 40a, 30a, 20a, 10a, a$ (top to bottom) where $a = 3.4$ Angstroms. Subfigure (a) shows results for lattice temperature $T_{\mathrm{L}} = 10$ K and Subfigure (b) for $T_{\mathrm{L}} = 50$ K.

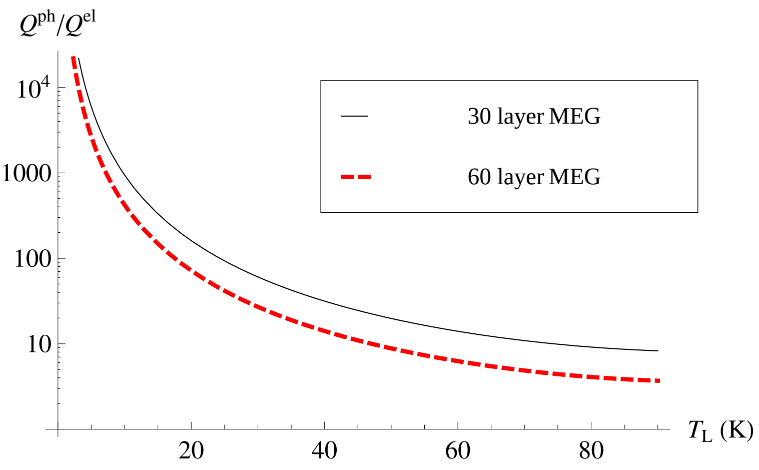

Supplementary Figure 4. **Acoustic phonon pinning of $T_{\rm HD}$ to $T_{\rm L}$.** Ratio of the acoustic phonon cooling power $\mathcal{Q}^{\rm ph}$ in the HD layers of MEG to the interlayer Coulombic energy transfer rate $\mathcal{Q}^{\rm el}$ from the LD to the HD layers of MEG as a function of the lattice temperature $T_{\rm L}$.

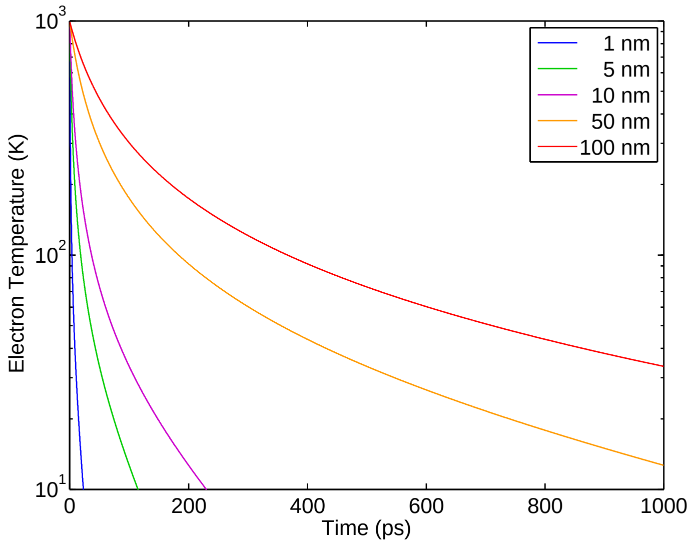

Supplementary Figure 5. **Disorder-assisted electron-phonon (supercollision) cooling in HD graphene.** Electron temperature dynamics $T(t) - T_{\mathrm{L}}$ predicted by the disorder-assisted electron-phonon cooling mechanism for HD graphene with $E_{\mathrm{F}} = 100$ meV at $T_{\mathrm{L}} = 10$ K for variable disorder mean free path l.

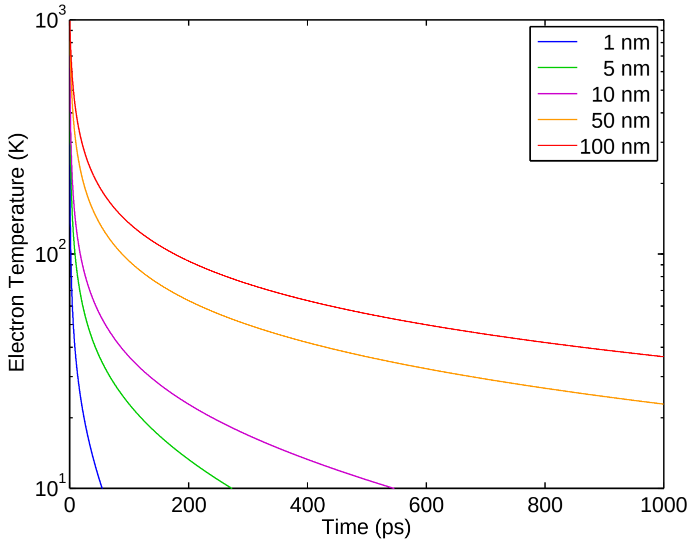

Supplementary Figure 6. **Disorder-assisted electron-phonon (supercollision) cooling in LD graphene.** Electron temperature dynamics $T(t) - T_{\mathrm{L}}$ predicted by the disorder-assisted electron-phonon cooling mechanism for LD graphene with $E_{\mathrm{F}} = 10$ meV at $T_{\mathrm{L}} = 10$ K for variable disorder mean free path l.

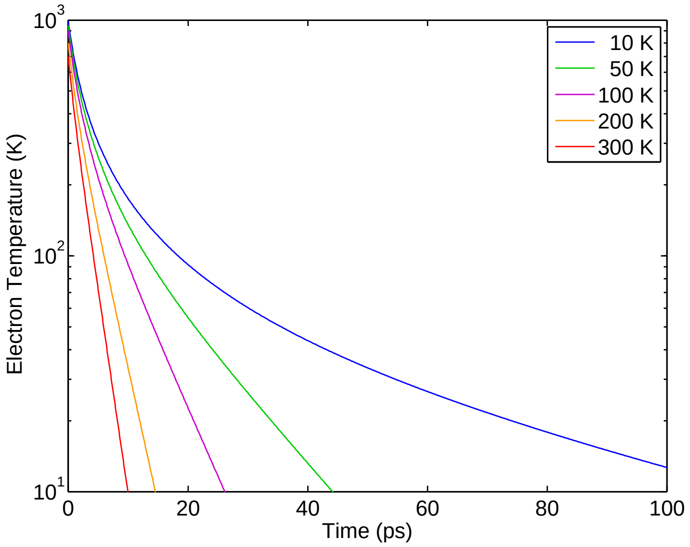

Supplementary Figure 7. **Disorder-assisted electron-phonon (supercollision) cooling in HD graphene.** Electron temperature dynamics $T(t) - T_{\mathrm{L}}$ predicted by the disorder-assisted electron-phonon cooling mechanism for HD graphene with $E_{\mathrm{F}} = 100$ meV and disorder mean free path $l = 5$ nm for variable T_{L}.

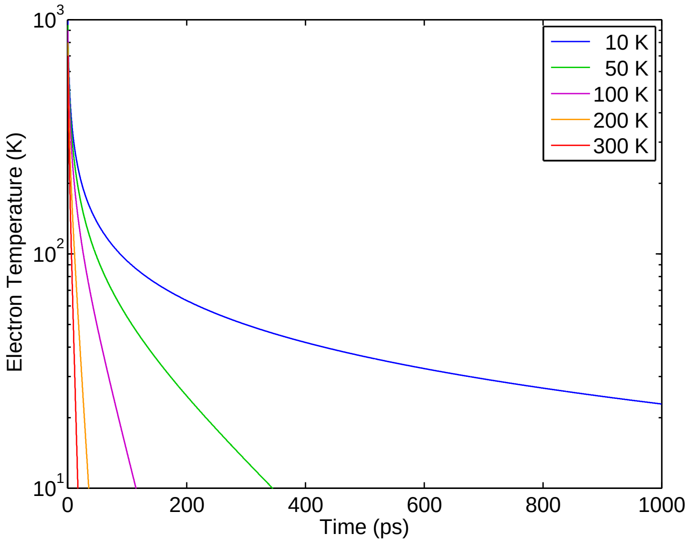

Supplementary Figure 8. **Disorder-assisted electron-phonon (supercollision) cooling in LD graphene.** Electron temperature dynamics $T(t) - T_{\mathrm{L}}$ predicted by the disorder-assisted electron-phonon cooling mechanism for LD graphene with $E_{\mathrm{F}} = 10$ meV and disorder mean free path $l = 50$ nm for variable T_{L}.

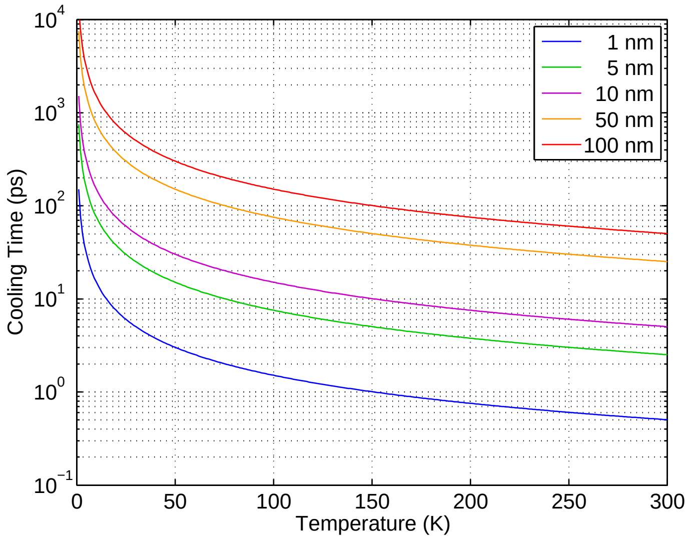

Supplementary Figure 9. **Disorder-assisted electron-phonon (supercollision) cooling in HD graphene.** Electronic cooling time τ_{HD} in the low electron temperature limit predicted by the disorder-assisted electron-phonon cooling mechanism for HD graphene with $E_{\mathrm{F}} = 100$ meV as a function of T_{L} for variable disorder mean free path l.

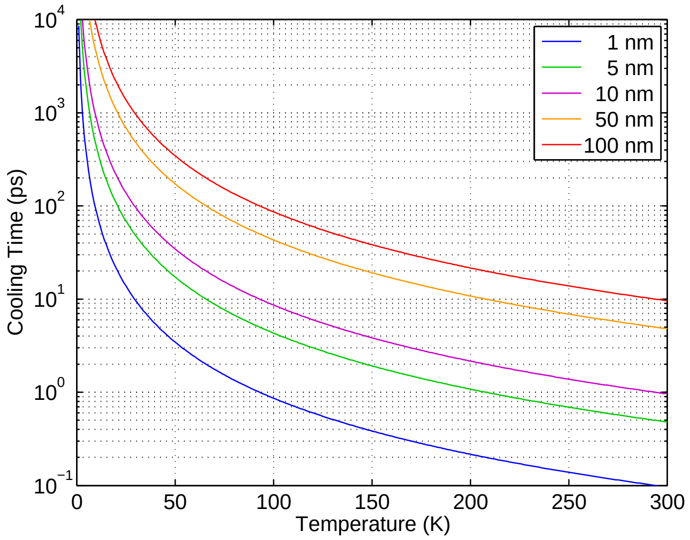

Supplementary Figure 10. **Disorder-assisted electron-phonon (supercollision) cooling in LD graphene.** Electronic cooling time τ_{LD} in the low electron temperature limit predicted by the disorder-assisted electron-phonon cooling mechanism for LD graphene with $E_{\mathrm{F}} = 10$ meV as a function of T_{L} for variable disorder mean free path l.

SUPPLEMENTARY NOTES

Supplementary Note 1. Normalized differential THz transmission spectra $\Delta t(\omega)/t(\omega)$

Here, we present additional experimental data to further demonstrate that the normalized differential THz transmission spectra are remarkably dispersionless in the detectable frequency range under all experimental conditions. Supplementary Figure 1 and Supplementary Figure 2 show the differential THz transmission spectra normalized to the THz transmission without photoexcitation, $\Delta t(\omega)/t(\omega)$, for variable substrate temperature and variable pump fluence, respectively, for a MEG sample with ~ 63 layers. The frequency-independent dynamic THz response of the MEG samples justifies recording the normalized differential THz transmission only at the peak of the THz probe pulse, $\Delta t/t$, as a function of pump-probe delay to map out the relaxation dynamics of the photoexcited carriers. The slight fluctuations in a few of the data scans for frequencies $\gtrsim 1.7$ THz are due to water vapor absorption arising from very slight fluctuations in the humidity level between the sample and the reference scans. We note that spectra obtained from Fourier-transformed time-domain measurements are much more susceptible to noise than direct spectrally-resolved measurements, because (long-term) fluctuations in the time domain are transferred into uncertainties in the frequency domain during the Fourier transformation process. On the other hand, direct time-resolved THz spectroscopy measurements of the relaxation dynamics are less prone to noise from fluctuations, and a very high signal-to-noise ratio (SNR) can be achieved through sufficient integration.

Supplementary Note 2. Interlayer energy transfer linearized in ΔT

Here, we derive an approximation for the interlayer Coulombic energy transfer rate between a lightly doped (LD) layer and a highly doped (HD) layer in MEG systems. We neglect electron tunneling between layers. Energy transfer between layers can nevertheless occur when an electron in one layer scatters off an electron in a remote layer. To first order in the temperature difference $\delta T = T_{\mathrm{LD}} - T_{\mathrm{HD}}$, we find that the energy transfer rate between thermal electron distributions in two Coulomb-coupled layers is given by:

$$\begin{aligned} \mathcal{Q}^{\mathrm{el}}_{ij} =& \frac{\hbar}{\pi}\int_{-\infty}^{\infty}\omega d\omega \sum_{\vec{q}} |v^{\mathrm{sc}}_{ij}|^2 \\ & \times \frac{\hbar\omega}{4T_{\mathrm{L}}^2}\frac{\delta T}{\sinh^2(\hbar\omega/2T_{\mathrm{HD}})} \\ & \times \mathrm{Im}[\chi_i(q,\omega,T_{\mathrm{HD}})]\mathrm{Im}[\chi_j(q,\omega,T_{\mathrm{HD}})]. \end{aligned} \tag{1}$$

Equation 1 can be derived by summing over all interlayer electron collision processes and combining a Fermi golden-rule expression for the transition rates with a random phase approximation expression for the electron-electron scattering amplitudes. We focus on the low temperature limit (i.e. $T \ll T_{\mathrm{F,LD}}, T_{\mathrm{F,HD}}$), where several physical approximations can be made. In this case, it is clear that the $\omega \to 0$ limit dominates the integrand of Equation 1. We note that in this low frequency limit, $\mathrm{Im}[\chi_i(q,\omega \to 0)] = -\nu_i\omega/(v_{\mathrm{F}}q)$, where $\nu_i = 2E_{\mathrm{F},i}/(\pi\hbar^2 v_{\mathrm{F}}^2)$ is the density of states at the Fermi energy. This linear dependence on frequency is also found in parabolic band 2DEG's, and describes how the particle-hole excitation spectrum vanishes with decreasing excitation energy $\hbar\omega$ [1]. Additionally, in the degenerate regime interlayer particle scattering allows a maximum change in electron wavevector of $\Delta q_{\mathrm{max}} = 2k_{\mathrm{F}}$, a fact reflected in:

$$\mathrm{Im}[\chi_i(q,\omega \to 0)] = 0, \qquad q > 2k_{\mathrm{F}}. \tag{2}$$

The interlayer Coulomb interaction, proportional to e^{-qd}, naturally places the limit $q \lesssim 1/d$, where d is the interlayer separation. However, $k_{\mathrm{F,HD}} \ll 1/d$ and we can approximately neglect interlayer separation (i.e. $d \to 0$) in Equation 1. Then, the MEG dielectric function reduces to a Thomas-Fermi-like form:

$$\epsilon^{\mathrm{MEG}}_{\mathrm{TF}}(q) = 1 + \frac{q^{\mathrm{MEG}}_{\mathrm{TF}}}{q}, \qquad q^{\mathrm{MEG}}_{\mathrm{TF}} = \frac{2\pi e^2}{\kappa}\sum_j \nu_j, \tag{3}$$

where ν_j is the density of states in the j'th HD layer and κ is the background dielectric function of a thin film on SiC ($\kappa \approx (10+1)/2 = 5.5$). Because of the large difference in carrier density between layers near and far from the substrate, allowed q values are much smaller than $q_{\rm TF}^{\rm MEG}$ and the screened interlayer interaction reduces to $(2\pi e^2)/(\kappa q_{\rm TF}^{\rm MEG})$.

The remaining integrals in Equation 1 contain a logarithmic divergence at zero wavevector. This is removed using a cutoff of $\omega/v_{\rm F}$ which reflects the zero value of $\mathrm{Im}[\chi(q,\omega)]$ above the intraband particle-hole continuum.

Finally, we find that the linearized interlayer energy transfer rate per area between a pair of graphene layers in a MEG system (where one is HD and the other LD) is:

$$\begin{aligned} \frac{1}{L^2} \cdot \mathcal{Q}^{\rm el}_{\rm HD,LD} =& \gamma (T_{\rm LD} - T_{\rm HD}) \left(\frac{\nu_{\rm LD}\nu_{\rm HD}}{(\sum_j \nu_j)^2} \right) \\ & T_{\rm HD}^3 \ln\left(\frac{T_{\rm F,LD}}{T_{\rm HD}} \right), \end{aligned} \tag{4}$$

where $\gamma = (8\pi^2/30)(k_{\rm B}^4/\hbar^3 v_{\rm F}^2)$. The density of states in the HD layer (LD layer) is denoted by $\nu_{\rm HD}$ ($\nu_{\rm LD}$). The sum over index j runs over all HD layers in the MEG system, accounting for the approximation that the dominant screening effect originates from the majority fraction of carriers in the HD layers (see the main text). $T_{\rm F,LD} = E_{\rm F,LD}/k_{\rm B}$ is the Fermi temperature of the LD layer. Equation 4 becomes exact in the degenerate limit $T \ll T_{\rm F,LD}, T_{\rm F,HD}$.

Supplementary Note 3. Interlayer energy transfer to leading order in T

Here, we derive in detail the leading order in temperature formula for the interlayer Coulombic energy transfer rate between one lightly doped (LD) layer of graphene and one highly doped (HD) layer of graphene. This follows similar steps as in the previous section, but without the assumption of infinitesimal temperature separation between the LD and HD layers. As described in the main text, the interlayer energy transfer rate per area between an LD and an HD layer is:

$$\begin{aligned}\frac{\mathcal{Q}^{\rm el}}{L^2} =&\frac{\hbar}{4\pi^3}\int_{-\infty}^{\infty}\omega\, d\omega\int d\vec{q}\,|v^{\rm sc}_{\rm LD,HD}|^2\,[n_{\rm B}(T_{\rm LD})-n_{\rm B}(T_{\rm HD})]\\ \times&\quad {\rm Im}[\chi_{\rm LD}(q,\omega,T_{\rm LD})]{\rm Im}[\chi_{\rm HD}(q,\omega,T_{\rm HD})],\end{aligned}\tag{5}$$

where $n_{\rm B}(T)$ is a Bose distribution function and $v^{\rm sc}_{\rm LD,HD}=v_q/\epsilon^{\rm RPA}(q,\omega,T_{\rm LD},T_{\rm HD})$ is the screened interaction between electrons in opposing layers within the random phase approximation (RPA). Given that the separation of the two layers is a distance d, the RPA dielectric function is given by:

$$\begin{aligned}\epsilon^{\rm RPA}(q,\omega,T_{\rm LD},T_{\rm HD}) =&(1-v_q\chi_{\rm LD}(q,\omega,T_{\rm LD}))(1-v_q\chi_{\rm HD}(q,\omega,T_{\rm HD}))\\ &-\; v_q^2e^{-2qd}\chi_{\rm LD}(q,\omega,T_{\rm LD})\chi_{\rm HD}(q,\omega,T_{\rm HD}),\end{aligned}\tag{6}$$

where $v_q=2\pi e^2/\kappa q$ and $\chi_i(q,\omega,T)$ is the temperature dependent non-interacting density-response function of the i'th layer of graphene. We next assume the temperature of the electrons in the HD layer is pinned to the lattice temperature, and approximate this as zero relative to the high temperature in the LD layer, i.e. $T_{\rm HD}=T_{\rm L}\to 0$. Relabeling $T_{\rm LD}$ as T we have:

$$\begin{aligned}\frac{\mathcal{Q}^{\rm el}}{L^2} =&\frac{\hbar}{4\pi^3}\int_{-\infty}^{\infty}\omega\, d\omega\int d\vec{q}\,|\frac{v_qe^{-qd}}{\epsilon^{\rm RPA}(q,\omega,T)}|^2\,n_{\rm B}(T)\\ \times&\quad {\rm Im}[\chi_{\rm LD}(q,\omega,T)]{\rm Im}[\chi_{\rm HD}(q,\omega)].\end{aligned}\tag{7}$$

In the degenerate limit, $k_{\rm B}T << E_{\rm F,LD}$, the Bose distribution function limits the important frequencies to approximately $\omega\leq E_{\rm F,LD}/\hbar$, and reveals that it is the leading order in ω which will yield the leading order in T. With this motivation we make use of the low frequency limit, ${\rm Im}[\chi_i(q,\omega\to 0)]=-\nu_i\omega/(v_{\rm F}q)$, where $\nu_i=2E_{{\rm F},i}/(\pi\hbar^2v_{\rm F}^2)$ is the density of states at the Fermi energy. Similarly, the dielectric function can be reduced to:

$$\epsilon^{\rm RPA}(q,\omega,T)\to(1+\frac{q^{\rm TF}_{\rm LD}}{q})(1+\frac{q^{\rm TF}_{\rm HD}}{q})-e^{-2qd}\frac{q^{\rm TF}_{\rm LD}q^{\rm TF}_{\rm HD}}{q^2},\tag{8}$$

where the Thomas-Fermi wavevector of the i'th layer is defined as $q_i^{\mathrm{TF}} = q v_q \nu_i$. Making use of the fact that in our MEG samples $k_{\mathrm{F,LD}} d << 1$ and $q_{\mathrm{HD}}^{\mathrm{TF}}/k_{\mathrm{F,LD}} >> 1$, we simplify the interlayer energy transfer rate per area to:

$$\frac{\mathcal{Q}^{\mathrm{el}}}{L^2} = \frac{E_{\mathrm{F,LD}}^4 \nu_{\mathrm{LD}}}{2\pi^2 v_{\mathrm{F}}^2 \hbar^3 \nu_{\mathrm{HD}}} \int_0^\infty \Omega d\Omega \int_0^\infty Q dQ \left(\frac{1}{e^{\Omega/t} - 1}\right) \left(\frac{\Omega}{Q}\right)^2, \tag{9}$$

where we have introduced the dimensionless wavevector, $Q = q/k_{\mathrm{F,LD}}$, the dimensionless frequency, $\Omega = \hbar\omega/E_{\mathrm{F,LD}}$, and the dimensionless temperature, $t = k_{\mathrm{B}}T/E_{\mathrm{F,LD}}$. The wavevector integration here diverges logarithmically. To remedy this, we identify that $\mathrm{Im}[\chi_{\mathrm{LD}}(q,\omega,0)]$ vanishes for $\omega > v_{\mathrm{F}} q$, which cuts off the wavevector integration for $Q < \Omega$. Carrying out the remaining integrals, we obtain the leading order in temperature energy loss rate per area of the LD electrons:

$$\frac{\mathcal{Q}^{\mathrm{el}}}{L^2} = -\frac{E_{\mathrm{F,LD}}^4 \pi^2 \nu_{\mathrm{LD}}}{15 v_{\mathrm{F}}^2 \hbar^3 \nu_{\mathrm{HD}}} t^4 \ln t. \tag{10}$$

Supplementary Note 4. Interlayer energy transfer with no LD-LD layer coupling

Here, we illustrate the distance dependence of the interlayer Coulombic energy transfer rate by calculating the temperature dynamics of an individual LD layer coupled to the HD layers in MEG systems, when we neglect energy transfer between pairs of LD layers. Although Coulomb coupling between LD layers is very strong [2], by temporarily forcing $\mathcal{Q}^{\rm el}_{\rm LD,LD'} \to 0$ we can gain insight into the distance dependence of the interlayer energy transfer rate. The temperature dynamics of each individual LD layer coupled to the HD layers are then independent and obey:

$$\partial_t T_{\rm LD} = \left(\sum_{j \in {\rm HD}} \mathcal{Q}^{\rm el}_{{\rm LD},j}(T_{\rm HD} = T_{\rm L}, T_{\rm LD}, d_{j,{\rm LD}}) \right) / \mathcal{C}_{\rm LD}. \tag{11}$$

If we also approximate the heat capacity in the LD layers by the neutral graphene formula $\mathcal{C}_{\rm LD} = 18\zeta(3)T^2_{\rm LD}/(\pi v^2_{\rm F})$, we obtain the results shown in Supplementary Figure 3, where we have used this formula to evaluate $T_{\rm LD}(t)$ for several different values of $d_{\rm HD,LD}$. All of these curves are calculated for the typical carrier density of the LD layers in MEG, measured in experiment, of $n_{\rm LD} = 10^{10}{\rm cm}^{-2}$. The slowest ($d_{\rm HD,LD} = 60$ layers) and fastest ($d_{\rm HD,LD} = 1$ layer) temperature relaxation curves provide upper and lower bounds, respectively, on the true relaxation time of *all* LD layers when interlayer energy transfer between pairs of LD layers is no longer neglected, i.e. $\mathcal{Q}^{\rm el}_{\rm LD,LD'} \neq 0$.

Supplementary Note 5. Acoustic phonon pinning of T_{HD} to T_{L}

Here, we present some simple calculations in support of our assumption that acoustic phonon cooling is capable of pinning the electronic temperature in the HD layers at the lattice temperature while these layers act as a heat sink for energy dissipation from the remaining hot LD layers. Heuristically, we also note that previous ultrafast optical spectroscopy experiments [3] have observed thermal relaxation times in the HD layers of MEG of ~ 10 ps, *much* faster than the relaxation times in the LD layers of $\sim 100 - 500$ ps that we report here.

Acoustic phonon cooling has previously been investigated in the context of hot-carrier cooling in disorder-free monolayer graphene [4, 5]. In these single layer systems, as a result of the relatively large optical phonon energy in graphene ($\hbar\omega_{\mathrm{op}} \approx 200$ meV [6]), acoustic phonons serve as the primary intrinsic cooling mechanism over a large temperature window extending up towards $T \sim 250$ K [4]. To estimate the ability of acoustic phonon cooling to take away the electronic energy that is Coulomb-transfered from LD to HD layers, we use Equation 14 in Bistritzer and MacDonald [4] to calculate the total energy transfer rate to the the lattice. Using the carrier density profile of the four most highly doped layers measured in experiment ($E_{\mathrm{F},1-4} = 360, 218, 140,$ and 93 meV), we find that $\mathcal{Q}^{\mathrm{ph}}_{\mathrm{HD}} = 9.09(T_{\mathrm{HD}} - T_{\mathrm{L}})$ W cm^{-2} can be transferred to the lattice via carrier-phonon scattering in the HD layers. We can calculate the ratio of $\mathcal{Q}^{\mathrm{ph}}$ to the interlayer energy transfer rate $\mathcal{Q}^{\mathrm{el}}$ from the N LD layers to these four HD layers using Equation 13 in the main text. Supplementary Figure 4 suggests that for both $N = 30$ and $N = 60$ layer MEG systems, the acoustic phonon cooling power is sufficient to keep the HD layers pinned to the lattice temperature while absorbing energy from the distant LD layers.

Supplementary Note 6. Disorder-assisted electron-phonon (supercollision) cooling

Here, we present an application of the recently proposed disorder-assisted electron-phonon (supercollision) cooling mechanism [7] to MEG. We investigate the qualitative and quantitative differences between disorder-assisted electron-phonon cooling in HD and LD graphene and we clearly demonstrate that this cooling mechanism cannot alone explain electronic cooling in high quality MEG.

The electron temperature dynamics of a single graphene layer in the framework of the disorder-assisted electron-phonon cooling mechanism is given by:

$$\partial_t T = \mathcal{Q}^{\mathrm{sc}}/\mathcal{C}, \tag{12}$$

where $\mathcal{Q}^{\mathrm{sc}} = \partial_t \mathcal{E}$ is the electronic cooling rate due to supercollisions, $\mathcal{C} = \partial_T \mathcal{E}$ is the electronic heat capacity and $\mathcal{E}$ is the electronic energy density. The electronic cooling rate due to supercollisions in the degenerate limit when $k_{\mathrm{B}}T \ll E_{\mathrm{F}}$ is given by [7]:

$$\mathcal{Q}^{\mathrm{sc}} = -A(T^3 - T_{\mathrm{L}}^3), \tag{13}$$

with a rate coefficient $A = 9.62 g^2 \nu^2(E_{\mathrm{F}}) k_{\mathrm{B}}^3/(\hbar k_{\mathrm{F}} l)$, where $g = D/\sqrt{2\rho v_{\mathrm{s}}^2}$ is the electron-phonon coupling constant and $\nu(E_{\mathrm{F}}) = E_{\mathrm{F}}/(2\pi\hbar^2 v_{\mathrm{F}}^2)$ is the density of states at the Fermi level per one spin and one valley flavor. The rest of the parameters are the Fermi velocity v_{F}, the sound velocity v_{s}, the deformation potential D, the mass density ρ and the disorder mean free path l. The electronic cooling rate in the non-degenerate limit when $k_{\mathrm{B}}T \gg E_{\mathrm{F}}$ is given by [8]:

$$\mathcal{Q}^{\mathrm{sc}} = -B(T^5 - T_{\mathrm{L}}^5), \tag{14}$$

with a rate coefficient $B = ((4k_{\mathrm{B}}^2)/(E_{\mathrm{F}}^2))A$. Because both the supercollision cooling rate and the electronic heat capacity have different functional dependence on the electron temperature at high and at low doping densities, we need to consider the two cases separately.

For HD graphene, the heat capacity is given by $\mathcal{C} = \alpha T = ((2\pi E_{\mathrm{F}} k_{\mathrm{B}}^2)/(3\hbar^2 v_{\mathrm{F}}^2))T$. By substituting this expression and Equation 13 in Equation 12, we obtain [9]:

$$\partial_t T = -\frac{A}{\alpha}\frac{T^3 - T_{\mathrm{L}}^3}{T}. \tag{15}$$

The electronic cooling timescale is governed by the rate coefficient $A/\alpha \propto E_{\mathrm{F}}/k_{\mathrm{F}} l \propto 1/l$. In the high and low electron temperature limits, Equation 15 reduces to:

$$\partial_t T = -\frac{A}{\alpha} T^2 \qquad for \qquad T \gg T_{\mathrm{L}}, \tag{16}$$

$$\partial_t T = -\frac{3A}{\alpha} T_{\mathrm{L}}(T - T_{\mathrm{L}}) \qquad for \qquad T \approx T_{\mathrm{L}}, \tag{17}$$

with solutions given by:

$$T(t) = \frac{T_0}{1 + \frac{A}{\alpha} T_0 t} \qquad for \qquad T \gg T_{\mathrm{L}}, \tag{18}$$

$$T(t) = T_{\mathrm{L}} + (T_0 - T_{\mathrm{L}}) \exp\left(-\frac{3A}{\alpha} T_{\mathrm{L}} t\right) \qquad for \qquad T \approx T_{\mathrm{L}}. \tag{19}$$

For LD graphene, the heat capacity is approximated by $\mathcal{C} = \beta T^2 = ((18\zeta(3)k_{\mathrm{B}}^3)/(\pi\hbar^2 v_{\mathrm{F}}^2))T^2$. By substituting this expression and Equation 14 in Equation 12, we obtain:

$$\partial_t T = -\frac{B}{\beta} \frac{T^5 - T_{\mathrm{L}}^5}{T^2}. \tag{20}$$

The electronic cooling timescale is governed by the rate coefficient $B/\beta \propto 1/k_{\mathrm{F}} l \propto 1/E_{\mathrm{F}} l$. In the high and low electron temperature limits, Equation 20 reduces to:

$$\partial_t T = -\frac{B}{\beta} T^3 \qquad for \qquad T \gg T_{\mathrm{L}}, \tag{21}$$

$$\partial_t T = -\frac{5B}{\beta} T_{\mathrm{L}}^2 (T - T_{\mathrm{L}}) \qquad for \qquad T \approx T_{\mathrm{L}}, \tag{22}$$

with solutions given by:

$$T(t) = \frac{T_0}{\sqrt{1 + 2\frac{B}{\beta} T_0^2 t}} \qquad for \qquad T \gg T_{\mathrm{L}}, \tag{23}$$

$$T(t) = T_{\mathrm{L}} + (T_0 - T_{\mathrm{L}}) \exp\left(-\frac{5B}{\beta} T_{\mathrm{L}}^2 t\right) \qquad for \qquad T \approx T_{\mathrm{L}}. \tag{24}$$

We observe that the different functional form of the cooling rate and the heat capacity at high and at low doping densities results in qualitatively different electron temperature relaxation dynamics. In the low electron temperature limit, in particular, the temperature dynamics follows an exponential form with a lattice temperature dependent characteristic time $\tau_{\mathrm{HD}} = ((3A/\alpha)T_{\mathrm{L}})^{-1} \propto l/T_{\mathrm{L}}$ for HD graphene, and with a lattice temperature dependent characteristic time $\tau_{\mathrm{LD}} = ((5B/\beta)T_{\mathrm{L}}^2)^{-1} \propto E_{\mathrm{F}} l/T_{\mathrm{L}}^2$ for LD graphene.

To obtain quantitative estimates of the full electron temperature dynamics predicted by the disorder-assisted electron-phonon cooling mechanism, we solve Equation 15 and Equation 20 numerically. In all calculations, we use $v_{\mathrm{F}} = 1 \times 10^6$ m s^{-1}, $v_{\mathrm{s}} = 2.1 \times 10^4$ m s^{-1}, $\rho = 7.6 \times 10^{-7}$ kg m^{-2} and $D = 20$ meV. Supplementary Figure 5 and Supplementary Figure 6 show the calculated electron temperature dynamics for HD graphene with $E_{\mathrm{F}} = 100$

meV and for LD graphene with $E_{\mathrm{F}} = 10$ meV, respectively, at $T_{\mathrm{L}} = 10$ K for variable disorder mean free path l. The large uncertainty in the value of the disorder length scale leads to a very large spread in the calculated electron temperature dynamics.

The experimental measurement of the precise value of the disorder mean free path between supercollisions is a challenging task and reliable estimates are lacking in the literature. One reliable way to characterize the degree of disorder which can potentially be related to supercollision cooling is directly from the width of the Dirac cone near the Dirac point in the graphene band structure as measured in high-resolution angle-resolved photoemission spectroscopy (ARPES). We use such recent ARPES measurements to estimate the disorder mean free path value appropriate for our MEG samples [10]. In Ref. 10 (Figure S1), correlation lengths of $\sim 1-3$ nm in exfoliated graphene and correlation lengths exceeding ~ 50 nm (limited *only* by the instrument resolution, but are expected to be even longer) in C-face MEG have been reported. Supplementary Figure 7 shows the calculated electron temperature dynamics for HD graphene with $E_{\mathrm{F}} = 100$ meV and disorder mean free path $l = 5$ nm for variable T_{L}. We note that these calculations are consistent with recent experimental results based on photocurrent measurements on HD chemical-vapor-deposited (CVD) graphene [9]. Supplementary Figure 8 shows the calculated electron temperature dynamics for LD graphene with $E_{\mathrm{F}} = 10$ meV and disorder mean free path $l = 50$ nm for variable T_{L}.

We also present the electronic cooling times in the low electron temperature limit predicted by the disorder-assisted electron-phonon cooling mechanism that are more straightforward to compare to the experiment. Supplementary Figure 9 and Supplementary Figure 10 show the calculated electronic cooling times τ_{HD} for HD graphene with $E_{\mathrm{F}} = 100$ meV and τ_{LD} for LD graphene with $E_{\mathrm{F}} = 10$ meV, respectively, as a function of T_{L} for variable disorder mean free path l. We observe that the predictions of the supercollision cooling model applied to the HD layers of MEG are inconsistent both in magnitude and lattice temperature dependence with the ultrafast time-resolved THz spectroscopy experiments. We also observe that the model predictions for the LD layers of MEG can roughly capture the order of magnitude and the lattice temperature dependence for some disorder mean free path, but not the layer number dependence. Because the quality of our MEG samples is expected to be even higher than we have conservatively assumed in these calculations, we conclude that the disorder-assisted electron-phonon (supercollision) cooling can provide a parallel cooling

channel, but it is not dominant in MEG.

SUPPLEMENTARY REFERENCES

[1] G. F. Giuliani and G. Vignale, Theory of the Electron Liquid (Cambridge University Press, Cambridge, 2005).

[2] O. Ilic, M. Jablan, J. D. Joannopoulos, I. Celanovic, H. Buljan and M. Soljacic, Near-field thermal radiation transfer controlled by plasmons in graphene, *Phys. Rev. B* **85**, 155422 (2012).

[3] D. Sun, C. Divin, C. Berger, W. A. deHeer, P. N. First and T. B. Norris, Hot carrier cooling by acoustic phonons in epitaxial graphene by ultrafast pump-probe spectroscopy, *Phys. Status Solidi C* **8** (4), 1194-1197 (2011).

[4] R. Bistritzer and A. H. MacDonald, Electronic cooling in graphene, *Phys. Rev. Lett.* **102**, 206410 (2009).

[5] W.-K. Tse and S. Das Sarma, Energy relaxation of hot Dirac fermions in graphene, *Phys. Rev. B* **79**, 235406 (2009).

[6] C. Faugeras, A. Nerriere, M. Potemski, A. Mahmoud, E. Dujardin, C. Berger and W. A. deHeer, Few-layer graphene on SiC, pyrolitic graphite, and graphene: A Raman scattering study, *Appl. Phys. Lett.* **92**, 011914 (2008).

[7] J. C. W. Song, M. Y. Reizer and L. S. Levitov, Disorder-assisted electron-phonon scattering and cooling pathways in graphene, *Phys. Rev. Lett.* **109**, 106602 (2012).

[8] A. Laitinen, M. Oksanen, A. Fay, D. Cox, M. Tomi, P. Virtanen and P. J. Hakonen, Electron-phonon coupling in suspended graphene: supercollisions by ripples, *Nano Lett.* **14**, 3009-3013 (2014).

[9] M. W. Graham, S.-F. Shi, D. C. Ralph, J. Park and P. L. McEuen, Photocurrent measurements of supercollision cooling in graphene, *Nat. Phys.* **9**, 103-108 (2013).

[10] J. Baringhaus, M. Ruan, F. Edler, A. Tejeda, M. Sicot, A. Taleb-Ibrahimi, Z. Jiang, E. Conrad, C. Berger, C. Tegenkamp and W. A. deHeer, Exceptional ballistic transport in epitaxial graphene nanoribbons, *Nature* **506**, 349-354 (2014).
